# Effects of a patient-derived de novo coding alteration of CACNA1I in mice connect a schizophrenia risk gene with sleep spindle deficits

**DOI:** 10.1038/s41398-020-0685-1

**Published:** 2020-01-23

**Authors:** Ayan Ghoshal, David S. Uygun, Lingling Yang, James M. McNally, Violeta G. Lopez-Huerta, Mario A. Arias-Garcia, David Baez-Nieto, Andrew Allen, Megan Fitzgerald, Soonwook Choi, Qiangge Zhang, Jen M. Hope, Karena Yan, Xiaohong Mao, Thomas B. Nicholson, Kazuo Imaizumi, Zhanyan Fu, Guoping Feng, Ritchie E. Brown, Robert E. Strecker, Shaun M. Purcell, Jen Q. Pan

**Affiliations:** 1 Stanley Center for Psychiatric Research, Broad Institute, Cambridge, MA USA; 2 Department of Psychiatry, VA Boston Healthcare System & Harvard Medical School, Boston, MA USA; 3 McGovern Institute for Brain Research, MIT, Cambridge, MA USA; 4 Novartis Institutes for BioMedical Research, 181 Mass Ave., Cambridge, MA 02139 USA; 5 Wyss Institute, Harvard University, Cambridge, MA USA; 6 Brigham and Women’s Hospital, Harvard Medical School, Boston, MA USA; 7 Present Address: Department of Neurodevelopment and Physiology, Institute of Cellular Physiology, National Autonomous University of Mexico, Mexico City, Mexico

**Keywords:** Physiology, Neuroscience, Schizophrenia

## Abstract

*CACNA1I*, a schizophrenia risk gene, encodes a subtype of voltage-gated T-type calcium channel Ca_V_3.3. We previously reported that a patient-derived missense de novo mutation (R1346H) of *CACNA1I* impaired Ca_V_3.3 channel function. Here, we generated Ca_V_3.3-RH knock-in animals, along with mice lacking Ca_V_3.3, to investigate the biological impact of R1346H (RH) variation. We found that RH mutation altered cellular excitability in the thalamic reticular nucleus (TRN), where Ca_V_3.3 is abundantly expressed. Moreover, RH mutation produced marked deficits in sleep spindle occurrence and morphology throughout non-rapid eye movement (NREM) sleep, while Ca_V_3.3 haploinsufficiency gave rise to largely normal spindles. Therefore, mice harboring the RH mutation provide a patient derived genetic model not only to dissect the spindle biology but also to evaluate the effects of pharmacological reagents in normalizing sleep spindle deficits. Importantly, our analyses highlighted the significance of characterizing individual spindles and strengthen the inferences we can make across species over sleep spindles. In conclusion, this study established a translational link between a genetic allele and spindle deficits during NREM observed in schizophrenia patients, representing a key step toward testing the hypothesis that normalizing spindles may be beneficial for schizophrenia patients.

## Introduction

Genetic analyses of large patient cohorts have identified multiple genomic loci associated with the risk of schizophrenia. Many of these risk genes encode proteins involved in calcium signaling including *CACNA1C*, *CACNB2*, and *CACNA1I* that may ultimately converge on a common disease mechanism^1,2^. *CACNA1I* encodes the pore-forming α1 subunit of voltage-gated calcium Ca_V_3.3 channels that is expressed in a subset of central nervous system neurons including GABAergic neurons of the thalamic reticular nucleus (TRN). In TRN neurons, Ca_V_3.3 channels mediate rebound burst firing in response to transient membrane hyperpolarization that recovers Ca_V_3.3 channels from inactivation^3,4^. The TRN receives excitatory input from both cortex and thalamocortical cells, while only sending inhibitory input to thalamocortical relay neurons. Rebound burst firing of the TRN mediates oscillatory activities in the thalamocortical circuitry, such as sleep spindles^5^. Sleep spindles are waxing and waning bursts of 8–15 Hz oscillations (sigma frequency band) detected by electroencephalogram (EEG) and represent a signature of stage 2 non-rapid eye movement (NREM) sleep in humans^6^. Spindle oscillations have been shown to be important for sleep-dependent memory consolidation in both mice^7^ and humans^8^, which underlie normal cognitive functioning.

Marked reductions of spindle activity during NREM have been reported in chronic as well as early course schizophrenia patients with otherwise normal sleep architecture^9^. While these reports typically involve relatively small sample sizes, spindle deficits throughout the course of schizophrenia, in treatment-naïve patients^10^, and in first-degree relatives^11^ implicate abnormal function of thalamocortical circuitry. This abnormality before the onset of the disease is consistent with the finding of reduced thalamic volume and altered thalamocortical connection in ultra-high-risk adolescents for schizophrenia^12,13^. Further, reduced sleep spindle activity in schizophrenia has been correlated with impaired sleep-dependent cognitive functions^14–18^. Taken together, these findings suggest that sleep spindle abnormalities in schizophrenic patients are a heritable intermediate phenotype that may underlie cognitive impairment. In this report, we aim to determine whether a patient-derived coding mutation (R1346H) in the schizophrenia-associated gene *CACNA1I* alters sleep spindle oscillations during NREM in vivo, to investigate potential translational markers across species.

Previous studies show that mice lacking Ca_V_3.3 exhibited a selective reduction in the power density of the sigma frequency band (defined as 10–12 Hz by the authors) only at transitions from NREM to rapid eye movement (REM) sleep in mice^19^ without any changes in sleep duration^20^. Sigma frequency analyses indirectly index spindle oscillation and do not detect individual spindles^21^. More critically, humans, unlike rodents, do not show the same increase in spindle activity just prior to NREM/REM transitions^6^. In order to address these questions and to investigate the impact of the R1346H mutation throughout NREM in vivo, we generated Ca_V_3.3 knock-out (KO, *Cacna1i*^*−/−*^ and *Cacna1i*^*+/−*^) and Ca_V_3.3-RH knock-in mice (RH, *Cacna1i*^*RH/RH*^ and *Cacna1i*^*+/RH*^), analyzed TRN neurophysiological function in acutely prepared brain slices, and quantified spindle oscillations using in vivo EEG recordings, explicitly considering spindle occurrence and morphology at 9, 11, 13, and 15 Hz. Here we report altered rebound bursting in TRN neurons and marked abnormalities in sleep spindles in both KO and RH knock-in mice, but not in KO heterozygous. These results highlighted genotype-specific consequences and establish a link between a genetic risk allele and a reduction in sleep spindles reported in schizophrenia patients, representing a key step toward testing the hypothesis that normalizing spindle-dependent impairments in schizophrenia patients are beneficial.

## Results

### Generation and validation of Ca_V_3.3 knock-in and knock-out mouse models

**Fig. 1:**
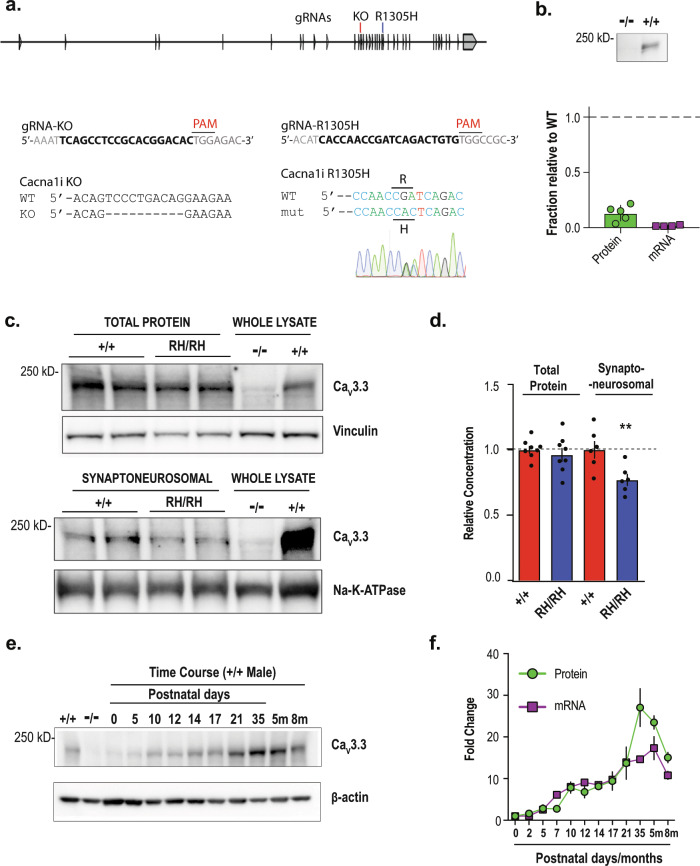
Generation and biochemical characterization of knock-in and knock-out animals. **a** The exon/intron structure of mouse *Cacna1i gene*, and the positions of targeting sgRNAs for generating the Ca_V_3.3 knock-out (KO) and R1305H knock-in animals. The sequences of the targeting sgRNAs and the genomic sequence verification for knock-out and knock-in founder animals are shown underneath. **b** Western blot from *Cacna1i*^*−/−*^ and *Cacna1i*^*+/+*^ whole-brain lysates showing a near complete lack of Ca_V_3.3 protein in the *Cacna1i*^*−/−*^ (−/−) samples. Significant reductions of both mRNA and protein levels in the heterozygous KO mice were also found (Supplementary Fig. 1b–d). Bottom panel: quantification of Ca_V_3.3 protein and mRNA levels normalized to *Cacna1i*^*+/+*^ levels (*n* = 5). **c** A typical representative western blot showing total Ca_V_3.3 protein levels (top panel) and Ca_V_3.3 protein levels in crude synaptoneurosomal preparations (bottom panel) from brain tissues derived from littermates of *Cacna1i*^*+/+*^ (+/+) and *Cacna1i*^*RH/RH*^ (RH/RH). **d** Quantification of total and synaptoneurosomal Ca_V_3.3 levels in the total brain lysate (*n* = 8) and crude synaptoneurosomal preparation (*n* = 6). **e** A typical western blot showing the developmental progression of Ca_V_3.3 expression in brain tissue across different postnatal days, and at 5 (5m) and 8 months (8m). **f** Quantification of protein (*n* = 2 for each time point) and mRNA (*n* = 2 for each time point except for 5m and 8m that contains *n* = 3 each) levels during developmental progression. **p < 0.01; error bars represent S.E.M.

Previously, we reported that R1346H, a de novo mutation identified in the schizophrenia proband by exome sequencing a cohort of trio samples, reduced the Ca_V_3.3 mediated Ca^2+^ currents in HEK293 cells^22^. In this report, we generated both knock-out (KO) and orthologous mutation R1305H knock-in (RH) Ca_V_3.3 mouse models using the CRISPR/Cas9-mediated genome editing approach coupled with pronuclear zygote injection (Fig. 1a, b)^23^. Loss of functional allele of Ca_V_3.3 was generated by pronuclear injection of Cas9 protein and an sgRNA, resulting in a mouse line with a 10 base-pair genomic deletion that causes a premature stop codon (Fig. 1a). The knock-in mutation was introduced through homologous recombination of a DNA template harboring the R1305H alteration (R1305H in murine Ca_V_3.3 corresponds to R1346H in human channel; Supplementary Fig. 1a), and subsequently sequence-confirmed in the founder animals. We confirmed the lack of Ca_V_3.3 wild-type (*Cacna1i*^*+/+*^) mRNA and channel protein in the homozygous KO animals (*Cacna1i*^*−/−*^; Fig. 1b), and significant reductions of both mRNA and protein levels in the heterozygous KO mice (*Cacna1i*^*+/−*^; Supplementary Fig. 1b–d). In the homozygous knock-in animals (*Cacna1i*^*RH/RH*^), the total amount of Ca_V_3.3 protein is similar to that of the *Cacna1i*^*+/+*^ mice (Fig. 1c, d; whole-cell lysate, Mann Whitney test; *p* = 0.65). However, the amount of Ca_V_3.3 channel protein was significantly reduced in the crude synaptoneurosomal preparation (Fig. 1c, d; synaptoneurosomal; Mann Whitney test; *p* = 0.0152) in the *Cacna1i*^*RH/RH*^ mice. This synaptoneurosomal preparation is enriched with dendrites and axonal processes including synaptic scaffolding protein PSD95 (Supplementary Fig. 1e). These results suggest that RH mutation does not impact overall channel protein production in neurons but may influence channel trafficking or localization in vivo, similar to previous analyses in human cell lines^22^.

Next, we characterized the expression of Ca_V_3.3 over developmental stages in the *Cacna1i*^*+/+*^ brain. As shown in Fig. 1e, f, the expression of Ca_V_3.3 mRNA transcripts and channel protein follow similar patterns where they were barely detectable at birth, started to increase around P10 and peaked around week 5. Such developmental expression profile coincides with the critical period of mouse thalamocortical development^24–26^, suggesting that Ca_V_3.3 may help shape key neuronal connections during development.

### Selective reduction of T-type Ca^2+^ currents in knock-in and knock-out mice

**Fig. 2:**
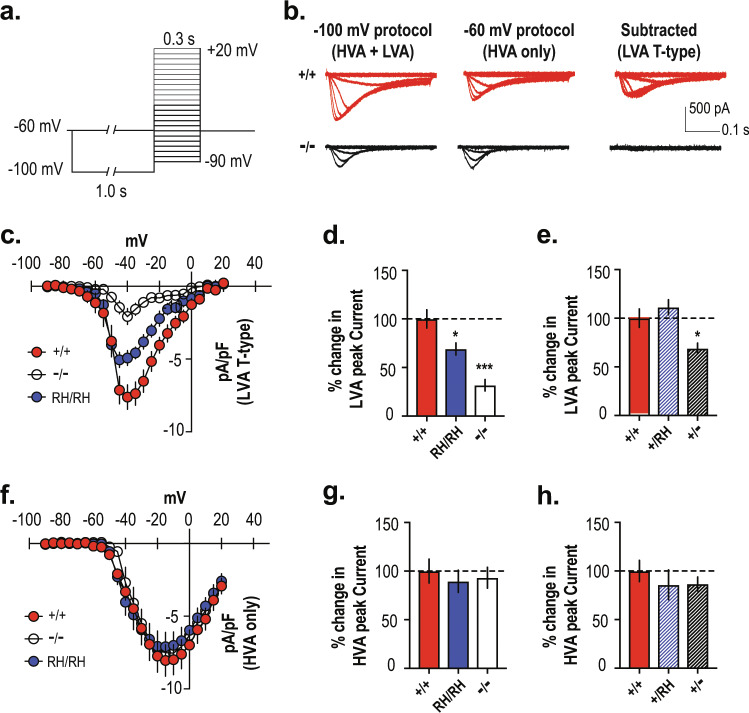
Reduction of T-type Ca^2+^ current in the *Cacna1i*^*RH/RH*^ and *Cacna1i*^*−/−*^ TRN neurons. **a** Protocol isolating T-type Ca^2+^ current in TRN neurons. Low-voltage-activated (LVA) together with high-voltage-activated (HVA) inward currents were elicited in neurons from a holding potential of −100 mV (for 1 s) to depolarizing test potentials ranging from −90 to +20 mV (Δ5 mV), in the presence of K^+^ and Na^+^ channel blockers AP-4 and TTX, respectively. HVA only inward currents were measured using the same protocol but from a holding potential of −60 mV. T-type Ca^2+^ currents from the TRN neurons were then isolated offline, by subtracting the HVA only inward current traces from the ones obtained from the HVA + LVA protocol. **b** Sample traces from *Cacna1i*^*+/+*^ and *Cacna1i*^*−/−*^ animals for voltage steps from −90 to −30 mV are shown for holding potentials at −100 and −60 mV, respectively, as well as the subtracted T-currents. While a significant amount of T-type Ca^2+^ current can be obtained after the subtraction method in the *Cacna1i*^*+/+*^ neuron, there was a complete lack of subtracted T-type currents in the *Cacna1i*^*−/−*^ neuron, validating the assay for T-type Ca^2+^ current isolation from mouse TRN neurons. **c** Mean current–voltage relationships (*I*–*V*) constructed for the subtracted T-type Ca^2+^ current density for *Cacna1i*^*+/+*^, *Cacna1i*^*RH/RH*^, and *Cacna1i*^*−/−*^ TRN neurons, showing a dramatic reduction in T-type Ca^2+^ current in the *Cacna1i*^*−/−*^ mice and a moderate reduction in peak current density in the *Cacna1i*^*RH/RH*^ mice. **d**, **e** Percent change in peak current density for T-type Ca^2+^ current is plotted from the homozygous (**d**; *p* < 0.0001; *R*^2^ = 0.563; *F* (2,32) = 2.181; one-way ANOVA) and heterozygous (**e**; *p* = 0.0027; *R*^2^ = 0.39; *F* (2,24) = 1.882; one-way ANOVA) TRN neurons, compared to *Cacna1i*^*+/+*^. **f** Mean *I*–*V* constructed for the HVA only Ca^2+^ current for *Cacna1i*^*+/+*^, *Cacna1i*^*RH/RH*^, and *Cacna1i*^*−/−*^ TRN neurons. **g**, **h** Percent change in HVA only Ca^2+^ current calculated for the homozygous (**g**; *p* = 0.80; *R*^2^ = 0.013; *F* (2,34) = 0.1574; one-way ANOVA) and heterozygous (**h**; *p* = 0.58; *R*^2^ = 0.0448; *F* (2,24) = 0.7681; one-way ANOVA) genotypes shows no significant differences in HVA Ca^2+^ current among the TRN neurons of all genotypes. *p < 0.05; ***p < 0.001; error bars represent S.E.M. All mean values and detailed statistics are listed in Supplementary Table 1.

Low-voltage-activated (LVA) and high-voltage-activated (HVA) calcium channels are present in TRN neurons^27–29^. T-type LVA Ca^2+^ channels are available to open at a holding potential of −100 mV but are inactivated at −60 mV where only HVA Ca^2+^ channels are available^30^. As shown in Fig. 2a, b, we isolated T-type Ca^2+^ currents by subtracting inactivation-resistant HVA Ca^2+^currents (pre-pulse of −60 mV) from the total amount of Ca^2+^ currents (pre-pulse of −100 mV). Using this protocol, we report a significant amount of T-type Ca^2+^ current in *Cacna1i*^*+/+*^ TRN neurons that exhibit maximum currents at −40 mV. This T-type inward current was mostly absent in *Cacna1i*^*−/−*^ animals, consistent with previous reports^3,19,31^. Similarly, the *Cacna1i*^*RH/RH*^ mice showed a notable reduction in the peak T-type Ca^2+^ current density (Fig. 2c, d; Supplementary Table 1), consistent with our previous analyses in cells^22^ and the reduced level of Ca_V_3.3 protein in the synaptoneurosomal preparation of knock-in animals (Fig. 1d). In a separate cohort, we found similar T-type channel peak current density in the *Cacna1i*^*+/+*^ and heterozygous knock-in (*Cacna1i*^*RH/+*^; Fig. 2e) mice. In contrast, heterozygous knock-out (*Cacna1i*^*+/−*^) mice displayed a significant reduction in the T-type current density (Fig. 2e; for means and *p* values see Supplementary Table 1), which is consistent with the biochemical analyses (Supplementary Fig. 1b–d). Importantly, there were no differences across all genotypes in the HVA inward current recorded, as is evident from the current–voltage relationship in *Cacna1i*^*+/+*^, *Cacna1i*^*RH/RH*^, and *Cacna1i*^*−/−*^ mice (Fig. 2f) as well as the percent change in peak current densities across the homozygous genotypes (Fig. 2g, Supplementary Table 1) or heterozygous genotypes (Fig. 2h, Supplementary Table 1). Together, these results indicate that there is a selective reduction in T-type Ca^2+^ conductance in the homozygous *Cacna1i*^*RH/RH*^, together with *Cacna1i*^*+/−*^ and *Cacna1i*^*−/−*^ mice without any apparent compensatory changes in the overall high-voltage-gated Ca^2+^ conductance in TRN neurons.

### Reduction of rebound burst firing in TRN neurons of Ca_V_3.3 knock-in and knock-out mice

**Fig. 3:**
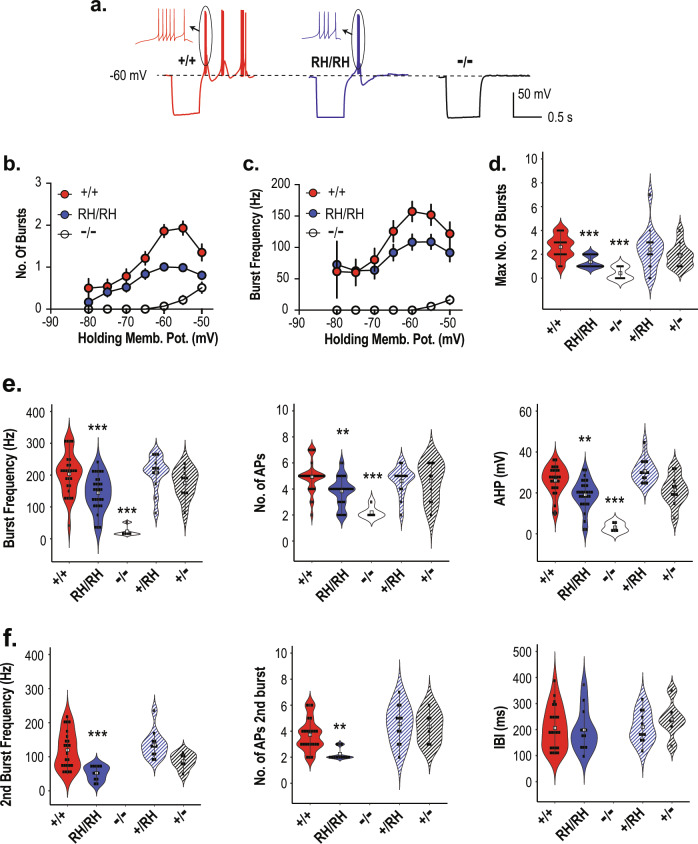
Reduction of rebound bursting in the *Cacna1i*^*RH/RH*^ and *Cacna1i*^*−/−*^ TRN neurons. **a** Sample traces from *Cacna1i*^*+/+*^, *Cacna1i*^*RH/RH*^, and *Cacna1i*^*−/−*^ mice showing hyperpolarization induced rebound burst firing in a representative TRN neuron from a holding potential of −60 mV. Inset displays a single burst from each genotype at high temporal resolution showing the classic acceleration–deceleration pattern. **b** Relationship of holding membrane potential and the number of rebound bursts observed in all TRN neurons of *Cacna1i*^*+/+*^, *Cacna1i*^*RH/RH*^, and *Cacna1i*^*−/−*^ animals. In general, *Cacna1i*^*+/+*^ neurons displayed increased number of bursts at more depolarized potentials. Lack of rebound bursting is apparent in *Cacna1i*^*−/−*^ TRN neurons, whereas *Cacna1i*^*RH/RH*^ TRN neurons show a clear reduction in peak number of rebound burst. **c** Relationship of holding membrane potential and the first burst frequency observed in TRN neurons of *Cacna1i*^*+/+*^, *Cacna1i*^*RH/RH*^, and *Cacna1i*^*−/−*^ animals. Similar to what is observed in **b**, in general there was an increase in first burst frequency with more depolarized holding potentials with an apparent reduction in peak burst frequency observed in *Cacna1i*^*RH/RH*^ neurons. There was also a dramatic reduction in burst frequency in the *Cacna1i*^*−/−*^ neurons that showed some form of rebound bursting. **d** The maximum number of bursts observed per neuron irrespective of holding potentials are plotted for all genotypes, showing a significant reduction in the *Cacna1i*^*RH/RH*^ and *Cacna1i*^*−/−*^ TRN neurons (*p* < 0.0001; *R*^2^ = 0.3836; *F* (4,88) = 13.69; one-way ANOVA). **e** First burst frequency (*p* < 0.0001; *R*^2^ = 0.4143; *F* (4,78) = 13.79; one-way ANOVA) and number of action potentials in the first burst (*p* < 0.0001; *R*^2^ = 0.2701; *F* (4,78) = 7.22; one-way ANOVA), as well as the after hyperpolarization (AHP; *p* < 0.0001; *R*^2^ = 0.4824; *F* (4,78) = 18.17; one-way ANOVA) observed in the first burst across all genotypes showing significant reductions in *Cacna1i*^*RH/RH*^ and *Cacna1i*^*−/−*^ animals. **f** Frequency (*p* < 0.0001; *R*^2^ = 0.361; *F* (3,48) = 9.04; one-way ANOVA), number of action potentials (*p* = 0.0001; *R*^2^ = 0.3529; *F* (3,48) = 8.73; one-way ANOVA) in the second burst, and the inter-burst-interval (IBI; *p* = 0.4027; *R*^2^ = 0.0246; *F* (3,48) = 0.4027; one-way ANOVA) between first and second burst across all genotypes showing significant reductions in *Cacna1i*^*RH/RH*^ TRN neurons. *Cacna1i*^*−/−*^ TRN neurons never showed repetitive bursting and are therefore excluded. **p < 0.01; ***p < 0.001; in each violin plot for **d**, **e** and **f** black squares represent individual data points and white boxes represent mean value; length of black line represents the interquartile range. All mean values and detailed statistics are listed in Supplementary Table 1.

Next, we characterized rebound burst firing and tonic firing in the TRN neurons of knock-in and knock-out mice (Fig. 3, Supplementary Table 1, Supplementary Fig. 2). A hyperpolarizing pulse of 500 ms removes the inactivation of T-type currents and induces oscillatory burst firing upon restoring to the holding potential in TRN neurons (Fig. 3a, *Cacna1i*^*+/+*^, red traces). These rebound bursts had the acceleration–deceleration pattern (Fig. 3a, inset) that was similar across all genotypes. The relationship between the holding potential and the number of bursts (Fig. 3b) or the action potential (AP) frequency of the first burst (Fig. 3c) is displayed across *Cacna1i*^*+/+*^, *Cacna1i*^*RH/RH*^ and *Cacna1i*^*−/−*^ genotypes. The mean and statistics for comparison are summarized in Supplementary Table 1. While such burst firing was largely absent in the *Cacna1i*^*−/−*^ mice (Fig. 3a, b, d), the *Cacna1i*^*RH/RH*^ showed an apparent reduction in the average number and frequency of the bursts between −65 and −50 mV (Fig. 3a–c), as well as the maximum number of bursts elicited (Fig. 3d). Notably, *Cacna1i*^*−/−*^ neurons showed occasional rebound burst firing at holding potentials more depolarized than −60 mV (Fig. 3b) and that these bursts from *Cacna1i*^*−/−*^ animals contained fewer APs, and with reduced frequency (Fig. 3b–e), indicating that such bursts in the *Cacna1i*^*−/−*^ neurons had different characteristics.

We further quantified the properties of rebound burst firing across the homozygous and heterozygous animals and compared to those in the wild-type *Cacna1i*^*+/+*^ mice (Fig. 3d–f). Significant reductions in the maximum AP frequency in the first burst (Fig. 3e, left panel), and the average number of APs in the first burst (Fig. 3e, middle panel) are present in the *Cacna1i*^*RH/RH*^ and *Cacna1i*^*−/−*^ mice, compared to *Cacna1i*^*+/+*^ mice. In the *Cacna1i*^*RH/RH*^ and *Cacna1i*^*−/−*^ TRN, we also observed reduced after hyperpolarization potential (AHP) of the first burst, which represents an indirect measure of Ca^2+^ mediated potassium efflux^32^ (Fig. 3e, right panel). No significant differences of these properties in heterozygous *Cacna1i*^*RH/+*^ and *Cacna1i*^*+/−*^ animals were observed compared to *Cacna1i*^*+/+*^ (Fig. 3e). In terms of repetitive bursting, whenever present, we further analyzed the frequency and the number of APs comprising the second burst across the genotypes (Fig. 3f). Significant reductions in the AP frequency and average number of APs were observed in the second burst of *Cacna1i*^*RH/RH*^ mice (Fig. 3f, left and middle panels). No statistical difference was observed in the second burst parameters for heterozygous animals (Cacna1i^+/RH^ and *Cacna1i*^*+/−*^) compared to *Cacna1i*^*+/+*^ animals. Interestingly, inter-burst interval (IBI) was similar across *Cacna1i*^*+/+*^, *Cacna1i*^*RH/RH*^, Cacna1i^+/RH^, and *Cacna1i*^*+/−*^ mice (Fig. 3f, right panel), suggesting that the Ca_V_3.3 channel may not contribute to the molecular mechanisms that regulate the IBI. Finally, there were no notable differences across the genotypes with respect to latency and threshold of APs of the rebound bursts or the passive membrane properties such as the input resistance and resting membrane potentials (Supplementary Table 1) in the TRN neurons.

We used 500 ms depolarization to induce tonic firing of TRN neurons (Supplementary Fig. 2a), and found no apparent difference in the current–frequency relationship (Supplementary Fig. 2b), the maximum firing frequency (Supplementary Fig. 2c), or the Ca^2+^ independent fast after hyperpolarization of APs (Supplementary Fig. 2d) across *Cacna1i*^*+/+*^, *Cacna1i*^*RH/RH*^, and *Cacna1i*^*−/−*^ TRN neurons. These results showed that tonic firing of TRN neurons remained largely intact under these recording conditions when Ca_V_3.3 function is impaired, consistent with intact HVA Ca^2+^ current in the mutant animals (Fig. 2f–h). Taken together, our data showed impaired rebound bursting, but not tonic firing, in the TRN neurons of *Cacna1i*^*−/−*^ and *Cacna1i*^*RH/RH*^ mice while heterozygous *Cacna1i*^*+/RH*^ and heterozygous *Cacna1i*^*+/−*^ mice are largely comparable to the wild-type *Cacna1i*^*+/+*^ mice in rebound bursting.

### Reduction of NREM sleep spindle density in knock-in and knock-out mice

In order to understand the physiological consequences of impaired rebound bursting, we recorded EEG activity in vivo and analyzed power spectral density and spindle activity during the light period (6 h), when nocturnal mice are predominantly asleep, across NREM, REM, and wake periods. In total, out of the 61 mice recorded, 56 mice (17 *Cacna1i*^*+/+*^, 10 *Cacna1i*^*+/−*^, 11 *Cacna1i*^*−/−*^, 8 *Cacna1i*^*+/RH*^, and 10 *Cacna1i*^*RH/RH*^) with at least one EEG channel that passed our quality control criteria (QC+, see Methods) were included in the analysis.

**Fig. 4:**
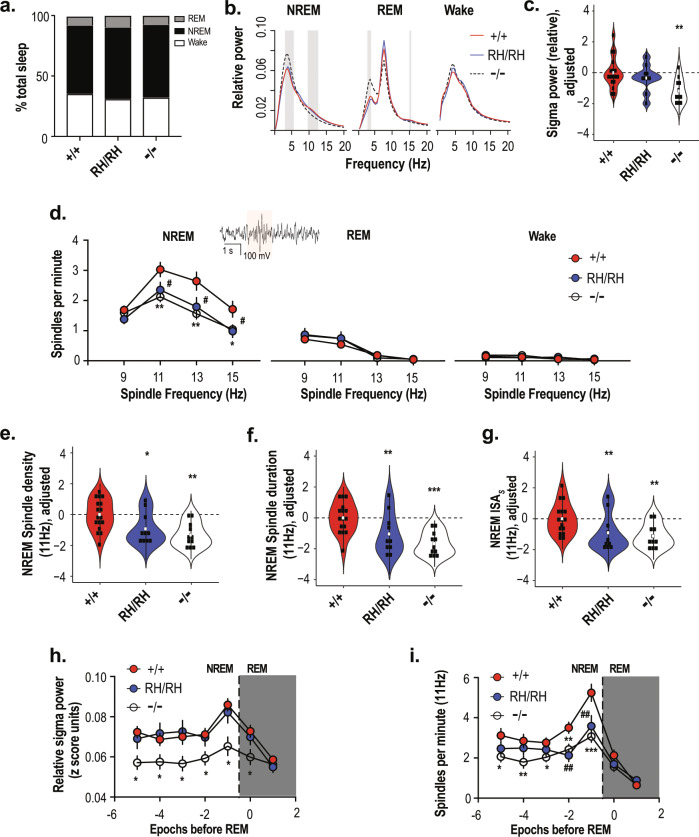
Reduction of sleep spindles in the *Cacna1i*^*RH/RH*^ and *Cacna1i*^*−/−*^ mice. **a** Sleep architecture showing similar proportion of time spent by *Cacna1i*^*+/+*^, *Cacna1i*^*RH/RH*^, and *Cacna1i*^*−/−*^ mice at the different stages of sleep during the 6 h light cycle analyzed. No changes were observed in sleep transitions as shown in Supplementary Table 2. **b** Power spectral density in *Cacna1i*^*+/+*^, *Cacna1i*^*RH/RH*^, and *Cacna1i*^*−/−*^ animals during NREM, REM, and wake periods of the 6 h light cycle. Spectral power at each frequency was normalized to total power and is expressed as relative power. Shaded areas show frequency at which significant differences were observed between *Cacna1i*^*+/+*^ and *Cacna1i*^*−/−*^ mice. No significant differences were observed between *Cacna1i*^*+/+*^ and *Cacna1i*^*RH/RH*^ mice. **c** Relative sigma power across *Cacna1i*^*+/+*^, *Cacna1i*^*RH/RH*^, and *Cacna1i*^*−/−*^ genotypes normalized to average *Cacna1i*^*+/+*^ values, showing a significant reduction in sigma power of *Cacna1i*^*−/−*^ animals. **d** NREM, REM, and wake average spindle density quantified as spindles per minute for *F*_C_ = 9, 11, 13, and 15 Hz spindles across *Cacna1i*^*+/+*^, *Cacna1i*^*RH/RH*^, and *Cacna1i*^*−/−*^ animals showing a significant reduction in sleep spindles only during NREM in both *Cacna1i*^*RH/RH*^ and *Cacna1i*^*−/−*^ animals compared to *Cacna1i*^*+/+*^. Inset shows a typical sleep spindle oscillation highlighted. # denotes statistical significance (*p* < 0.05) between *Cacna1i*^*+/+*^ and *Cacna1i*^*RH/RH*^, and * (*p* < 0.05) and ** (*p* < 0.01) denote statistical significance between *Cacna1i*^*+/+*^ and *Cacna1i*^*−/−*^ animals. **e** Spindle density for *F*_C_ = 11 Hz is normalized to *Cacna1i*^*+/+*^ average. *Cacna1i*^*RH/RH*^ and *Cacna1i*^*−/−*^ animals show significant reductions in the spindle density during NREM sleep. **f**, **g** Characteristics of *F*_C_ = 11 Hz spindles including duration (**f**) and integrated spindle activity per spindle (ISA_s_; **g**), normalized to the *Cacna1i*^*+/+*^ average values show that in addition to reductions in sleep spindle there is also significant reduction *Cacna1i*^*RH/RH*^ and *Cacna1i*^*−/−*^ spindle duration and ISA_s_. **p < 0.01; ***p < 0.001; in each violin plot **c**, **e**, **f** and **g** black squares represent individual data points and white boxes represent mean value; length of black line represents the interquartile range. **h** Relative sigma power in NREM (with each point representing the average of two consecutive 10-s epochs) preceding an REM epoch indicates a transient increase during NREM–REM transition (dashed vertical line) in *Cacna1i*^*+/+*^. Significant reduction in sigma power of *Cacna1i*^*−/−*^ animals is observed across all NREM epochs. **i** Average spindle density of *F*_C_ = 11 Hz spindles for NREM (with each point representing the average of two consecutive 10-s epochs) preceding an REM episode, showing that similar to sigma power there is an increase in mean spindle density during NREM–REM transition (dashed vertical line). Significant reduction of spindle density is observed in both *Cacna1i*^*RH/RH*^ and *Cacna1i*^*−/−*^ animals during the transition, although no changes in sigma power is observed in the *Cacna1i*^*RH/RH*^ mice (**d**). # (*p* < 0.05) and ## (*p* < 0.01) denote statistical significance between *Cacna1i*^*+/+*^ and *Cacna1i*^*RH/RH*^ and * (*p* < 0.05), ** (*p* < 0.01), and *** (*p* < 0.001) denote statistical significance between *Cacna1i*^*+/+*^ and *Cacna1i*^*−/−*^ animals. Error bars denote S.E.M. All mean values and detailed statistics are listed in Supplementary Table 3.

Genotype groups were broadly similar for sleep stage duration (Fig. 4a) and rates of stage transitions (Supplementary Table 2), and the proportion of epochs removed by automated artifact detection, suggesting comparable sleep architecture.

#### EEG power spectral density

During wakefulness, we did not observe differences in relative EEG power spectral density among genotypes, for any frequency tested (0.5–20 Hz in 0.5 Hz bins; Fig. 4b). In contrast, during NREM we observed significantly (*p* < 0.01) reduced power in the sigma range, with a maximal difference at 11 Hz, in *Cacna1i*^*−/−*^ mice compared to *Cacna1i*^*+/+*^ (Fig. 4b, c), whereas the homozygous knock-in mice (*Cacna1i*^*RH/RH*^) were similar to *Cacna1i*^*+/+*^. Sigma (11–15 Hz) band relative power was also reduced in *Cacna1i*^*−/−*^ mice compared to *Cacna1i*^*+/+*^ (*p* = 0.005; Fig. 4c), whereas *Cacna1i*^*+/−*^ (*p* = 0.13), *Cacna1i*^*+/RH*^ (*p* = 0.39), and *Cacna1i*^*RH/RH*^ (*p* = 0.86) groups were similar to *Cacna1i*^*+/+*^ (heterozygous mice data displayed in Supplementary Fig. 3a). In addition to reduced sigma power, *Cacna1i*^*−/−*^ mice had increased power at lower frequencies, in particular delta range (3–5 Hz, Fig. 4b, *p* = 0.001); heterozygous *Cacna1i*^*+/−*^ mice also showed a small increase in this frequency range (*p* = 0.044) relative to *Cacna1i*^*+/+*^ (Supplementary Fig. 4). Neither *Cacna1i*^*+/RH*^ (*p* = 0.92) nor *Cacna1i*^*RH/RH*^ (*p* = 0.83) mice exhibited increased delta frequency oscillation power. Finally, the increase in NREM delta frequency observed in *Cacna1i*^*−/−*^ was also present during REM, as was the decrease in sigma power, albeit to a lesser extent (Fig. 4b).

#### Spindle detection

We next applied a wavelet-based algorithm to detect spindles directly, centering on four target frequencies (*F*_C_ = 9, 11, 13, and 15 Hz). Spindle density (counts per minute) was only modestly correlated with relative sigma power (e.g. during NREM, *r* = 0.25, *p* = 0.06 for *F*_C_ = 13 Hz spindles). As expected, when detecting spindles across all stages with similar detection parameters, we observed markedly lower spindle densities during REM sleep and wake compared to NREM sleep, across all groups and target frequencies (Fig. 4d). For example, *F*_C_ = 11 Hz spindle density in *Cacna1i*^*+/+*^ mice was 3.02 spindles per minute during NREM compared to 0.55 during REM and 0.12 during wakefulness (Supplesmentary Table 3).

Compared to wild type, both *Cacna1i*^*−/−*^ and *Cacna1i*^*RH/RH*^ groups showed large (−0.9 and −0.66 standard deviation units, respectively, for *F*_C_ = 11 Hz spindles) statistically significant reductions in spindle density during NREM (Fig. 4d, e, the mean and statistical comparison are summarized in Supplementary Table 3). These genotype-associated differences were strongest for *F*_C_ = 11 Hz spindles (*p* = 0.002 and *p* = 0.027; Fig. 4e) although broadly similar effects were observed for *F*_C_ = 13 Hz spindles (*p* = 0.002 and *p* = 0.025) and 15 Hz spindles (*p* = 0.011 and *p* = 0.028) (Fig. 4d, Supplementary Fig. 5), but not slower *F*_C_ = 9 Hz spindles (*p* = 0.17 and *p* = 0.14; Supplementary Fig. 5). Analyses restricted to *stable* epochs (those not contiguous with a stage transition, i.e. NREM epochs flanked by other NREM epochs) yielded similar results. In contrast, no significant differences in NREM spindle density were observed for the *Cacna1i*^*+/−*^ and Cacna1i^+/RH^ mice compared to *Cacna1i*^*+/+*^ (Supplementary Figs. 5 and 6), which is consistent with the wild-type-like rebound bursting in TRN neurons of the heterozygous animals (Fig. 3d–f).

In contrast to NREM, there were no significant differences in spindle densities among genotypes during REM and wake periods when focusing on stable REM and wake epochs not flanking stage transitions (Supplementary Table 3). Thus, the spindle deficits observed in both *Cacna1i*^*−/−*^ and *Cacna1i*^*RH/RH*^ mice appear to be specific to NREM and consistent with the spectral analyses in Fig. 4b.

#### Spindle density at NREM–REM transitions

Relative to stable NREM sleep, rodents show a characteristic peak^19,33^ of sigma and spindle activity approximately 30 s prior to NREM/REM transitions, followed by an immediate reduction in sigma power and spindling during subsequent REM (Fig. 4h, i). As expected in *Cacna1i*^*+/+*^ mice, we observed these peak activities of both sigma and spindle density during NREM/REM transtion, for all values of *F*_C_ (Fig. 4h, i, Supplementary Fig. 7). The reductions in spindle density described above, for *Cacna1i*^*−/−*^ and *Cacna1i*^*RH/RH*^ mice during all NREM, were pronounced prior to NREM/REM transitions (Fig. 4i). Analyzed in 20-s intervals (i.e. pairs of adjacent epochs), *Cacna1i*^*−/−*^ and *Cacna1i*^*RH/RH*^ mice showed reductions (*p* = 0.007 and *p* = 0.001, respectively, for the second interval (30–50 s) prior to the transition; *p* = 0.0002 and *p* = 0.006 for the first interval (10–30 s) prior) in F_C_ = 11 Hz spindle density compared to *Cacna1i*^*+/+*^. Standardizing densities to have 0 mean and unit variance within the *Cacna1i*^*+/+*^ group, the means for *Cacna1i*^*−/−*^ and *Cacna1i*^*RH/RH*^ mice were −2.8 and −1.8 SD units, respectively, indicative of very large effect sizes. As before, heterozygous mice failed to show any differences in spindle density prior to NREM–REM transitions (Supplementary Fig. 7).

#### Genotype differences in NREM spindle morphology

In addition to changes in spindle density, homozygous knock-in and knock-out mice also showed notable differences in spindle morphology. For *F*_C_ = 11 Hz NREM spindles, *Cacna1i*^*−/−*^ and *Cacna1i*^*RH/RH*^ groups exhibited significantly reduced spindle durations (Fig. 4f; *p* = 1×10^−5^ and *p* = 0.001, respectively; Supplementary Table 4) and spindle-wise measures of integrated spindle activity (ISA_s_, reflecting both spindle duration and amplitude; Fig. 4g; *p* = 0.001 and *p* = 0.004; Supplementary Table 4). That is, beyond differences in the number of spindles detected, those that were detected were significantly smaller in *Cacna1i*^*−/−*^ and *Cacna1i*^*RH/RH*^ mice compared to *Cacna1i*^*+/+*^. No changes in the spindle (*F*_C_ = 11 Hz) duration or ISA_s_ were observed in heterozygous *Cacna1i*^*+/−*^ and *Cacna1i*^*+/RH*^ mice (Supplementary Fig. 8).

In summary, both *Cacna1i*^*−/−*^ and *Cacna1i*^*RH/RH*^ mice showed marked deficits in spindle density and morphology compared to wild-type mice, while no significant changes were observed in the heterozygous knock-out and knock-in mice (Supplementary Fig. 3). Major genotype-dependent differences in spindle activity were specific to NREM sleep, with no clear pattern of significant effects during REM or wake. While most pronounced just prior to NREM/REM transition, these spindle deficits were significant when considering all NREM in the sleep period. Importantly, the deficits in *Cacna1I*^*RH/RH*^ mice were only revealed by analyses that directly detected and characterized individual spindles, rather than traditional spectral analyses based on average sigma power.

## Discussion

*CACNA1I* has been implicated by both genome-wide common variant association^2^ and de novo exonic variant analyses^34^ as a genetic risk factor for schizophrenia. However, its connection to the disease is elusive. This study generated mice harboring the de novo mutation identified in a schizophrenic patient (*Cacna1i*^*RH/RH*^) and investigated its biological impact. We found T-type Ca^2+^ current density was significantly reduced in the TRN neurons of the *Cacna1i*^*RH/RH*^ mice, without compensatory changes in high voltage activated Ca^2+^ currents (Fig. 2), lending confidence in addressing Ca_V_3.3-specific function in these mice. The reduction in Ca_V_3.3 currents led to robust functional deficits in TRN excitability (Fig. 3), and impaired spindle oscillations in vivo (Fig. 4).

While the previous study on Ca_V_3.3 KO mice reported sigma power deficits during NREM/REM transition^19^, this report demonstrated sleep spindle occurrences and morphology deficits throughout NREM as well as during NREM/REM transition in mice harboring the RH mutation and lacking Ca_V_3.3 (Fig. 4), thus directly connecting *CACNA1I* to sleep spindle deficits reported in schizophrenia patients^9–11,16–18^ during NREM. Given that humans, unlike rodents, do not show the same increase in spindle activity just prior to NREM/REM transitions^6^, our new findings strengthened the inferences we can make on spindle oscillation between species.

Whereas no significant deficits in sigma power were observed in *Cacna1i*^*RH/RH*^ mice, our automated spindle detection algorithm revealed a robust deficit in sleep spindle density through NREM sleep and immediately prior to NREM/REM transitions. Unlike sigma power analyses, which assumes statistical stationarity of time series and captures tonic/baseline sigma activity, the spindle detection explicitly recapitulates the definition of a spindle as a temporally limited, non-stationary burst of increased, phasic activity relative to the baseline. Thus, it can be expected that spindle density may better capture transient changes in sigma activity (i.e. discrete and relatively obvious spindle events) compared to spectral power statistics computed across a longer time period, as seen in human EEG analyses^6^. On the other hand, sigma power may better capture activity related to very low amplitude or short spindles that are missed by our current thresholding factor needed to detect spindles as discrete events. There was no alteration in the overall sleep architecture or sleep transitions between sleep stages, during the light cycle of the mutant mice, indicating loss or reduction of Ca_V_3.3 function may not directly shape the onset of sleep stages. Indeed, the larger human studies looking at sleep spindles in schizophrenia^16^ did not observe any significant differences in macro-architecture between cases and controls, but did observe differences in spindle activity. This shows that the Ca_V_3.3 model can be utilized to understand consequences of specific deficits in spindle oscillations during sleep.

Our results across homozygous and heterozygous mice consistently show that when there is a significant deficit in rebound bursting, there is a deficit in sleep spindle density (Figs. 3 and 4; Supplementary Figs. 5 and 6), suggesting that rebound bursting correlates and predicts sleep spindle deficits during NREM sleep in vivo in these mice. Comparing the heterozygous *Cacna1i*^*+/−*^ and the homozygous *Cacna1i*^*RH/RH*^ animals, it is unclear why both models produced reduced T-type Ca^2+^ current density (Fig. 2), yet only *Cacna1i*^*RH/RH*^ model had TRN rebound bursting deficits (Fig. 3). One explanation for such discrepancy may lie in the subcellular location of the Ca_V_3.3 channels, as higher Ca_V_3.3 density in distal dendrites^3^ is critical for rebound bursting. It may be possible that the Ca_V_3.3 channel density in the distal dendrites of the *Cacna1i*^*+/−*^ mice is comparable to wild-type mice despite reduced mRNA levels, whereas that in the *Cacna1i*^*RH/RH*^ mice is much reduced and unable to support normal rebound bursting. This effect induced by the RH mutation may be caused by certain deficits such as trafficking impairment as suggested previously by our work in human cell lines^22^. Further analyses in Ca_V_3.3 trafficking in vivo and dendritic excitability of TRN neurons could test this hypothesis. Regardless of mechanism, differential rebound bursting patterns in *Cacna1i*^*RH/RH*^ and *Cacna1i*^*+/−*^ animals indicating that simple Ca_V_3.3 haploinsufficiency alone is not enough to induce the rebound bursting, or spindle deficits in mice. Furthermore, sigma frequency power analyses can detect deficits in homozygous KO animals but not in *Cacna1i*^*RH/RH*^ mice (Fig. 4), suggesting differences in spindling between the two genotypes.

Taken together, these results indicate that spindle activity may serve as an in vivo indicator for TRN function in mice, and that *Cacna1i*^*RH/RH*^ animals would allow studies that could not be done in *Cacna1i*^*+/−*^ *or Cacna1*^*−/−*^. For example, the ability of pharmacological or genetic perturbations on or upstream of Ca_V_3.3 to normalize the spindle deficits can only be tested in *Cacna1i*^*RH/RH*^ but not in *Cacna1i*^*+/−*^ (that do not show spindle deficits) or *Cacna1*^*−/−*^ (that shows spindle deficits but do not have the Ca_V_3.3 channel), as the Ca_V_3.3 channel is required for rebound burst firing in TRN neurons^19,31^ underlying sleep spindle oscillations. As such, *Cacna1i*^*RH/RH*^ provide a valuable model not only to dissect potential disease mechanism and spindle biology but also to evaluate pharmacological and genetic modulations.

Spectral analyses of sigma power have often been used in studies of sleep spindle neurocircuitry, as a surrogate index of spindle activity. For example, in three mouse studies, overall NREM sigma power was unchanged across EEG recording sessions when comparing experimental groups of mice, whereas it differed prior to NREM/REM transitions^19,35,36^. In contrast, our primary analyses of sleep employed a spindle detection pipeline based on a wavelet-based algorithm^6^, enabling us to quantify the density, morphology, and oscillation frequencies of individual sleep spindles at single Hertz resolution. As described in human EEG studies^6^, faster or slower spindles can exhibit different properties and functional associations. Several observations support the validity of our spindle detection procedure. First, we observed a markedly higher incidence of spindles during NREM sleep, whereas spindle-like oscillations were rarely detectable during REM periods and wakefulness during the light cycle (Fig. 4). Second, in accordance with previous reports in rodents^33,37,38^, we observed peak spindle density around 11 Hz and a steep increase in NREM spindle density immediately preceding a transition to REM (Fig. 4). Third, as would be predicted, based on the previous observation of reduced sigma power in *Cacna1i*^*−/−*^ mice just before NREM/REM transitions^19^, we observed a sharp reduction in spindle density in *Cacna1i*^*−/−*^ mice prior to NREM–REM transitions in addition to a reduction in sigma power.

Quantifying discrete spindle events may index TRN dysfunction more sensitively compared to average sigma band power. In our study, we observed spindle deficits not just at NREM/REM transitions in *Cacna1i*^*−/−*^ mice, but across all NREM sleep in both *Cacna1i*^*−/−*^ mice and *Cacna1i*^*RH/RH*^ mice, while analyses of sigma power alone did not differentiate *Cacna1i*^*RH/RH*^ animals. Whereas drastic manipulations of the circuit—like the total loss of rebound bursting in *Cacna1i*^*−/−*^ mice—can lead to noticeable differences in the overall sigma power (Figs. 3 and 4), more subtle changes—such as reduced rebound bursting in *Cacna1i*^*RH/RH*^ mice —may impact TRN function that is not reflected in overall sigma power. These results highlight the importance of individual sleep spindle detection.

We show here that the *CACNA1I* risk variant induces spindle deficits, which corroborated with two recent studies of PTCHD1 and GRIA1, two genes implicated by neuropsychiatric genetics, that abnormal sleep spindle oscillation may underlie neurodevelopmental disorders such as autism and schizophrenia^39–41^. Moreover, the immunoreactivity of parvalbumin (PV) was found to be drastically reduced in the postmortem TRN neurons of schizophrenia and bipolar patients^40^, lending support for altered TRN function in schizophrenia pathophysiology. Several clinical studies reported reduced sleep spindle density in schizophrenia patients, drug-naive patients, and first-degree relatives of patients^9^, suggesting that sleep spindle impairments be an endophenotype in schizophrenia and are correlated with sleep-dependent cognitive impairement^14^. Clearly, behavioral tests of these *Cacna1i* genetic models should be next investigated to address the question of behavioral correlates of spindle deficits. In particular, sleep-dependent learning or phenotypes would be a focus of next-stage investigations. Nonetheless, our results from the *Cacna1i*^*RH/RH*^ mice linked a risk variant in schizophrenia with a quantifiable EEG endophenotype marker in NREM reported in schizophrenia patients, providing reverse and forward translational opportunities to understand deficits of sleep spindle and TRN function in schizophrenia^6,9^ and serving as a key step toward testing the therapeutic hypothesis that normalizing sleep spindles by targeting Ca_V_3.3 and/or TRN function may lead to novel strategies for treating schizophrenia patients.

## Methods

All methods are listed in the Supplementary Methods section. The spindle analysis pipeline and a schematic of wavelet-based spindle detection are illustrated in Supplementary Fig. 9 and Supplementary Fig. 10 respectively.

## Supplementary information

Al Supp Materials Merged
