# Supplementary material for: Effects of a patient-derived de novo coding alteration of CACNA1I in mice connect a schizophrenia risk gene with sleep spindle deficits
Source: Transl Psychiatry. 2020 Jan 23;10:29. doi: 10.1038/s41398-020-0685-1 (PMC7026444; doi:10.1038/s41398-020-0685-1)
Supplement: Supplementary file 1 — Al Supp Materials Merged [file 41398_2020_685_MOESM1_ESM.pdf]

**Generation of mutant mice and genomic validation:**

Two sgRNAs targeting each KO and RH site were designed using the Zhang lab's online CRISPR design tool (http://tools.genome-engineering.org) and cloned into pSpCas9(BB)[1]. Their effects were initially evaluated by transfecting this plasmid, which expresses both the guide RNA and Cas9, into the N2A mouse neuroblastoma cell line. The on-target cutting efficiency was evaluated by the SURVEYOR assay as described[1]. The final sgRNA for targeting each site was then chosen based on the results from N2A cells. The sgRNAs (sequence for KO: 5'-TCAGCCTCCGCACGGACAC-3' and RH 5'- CACCAACCGATCAGACTGTG -3') were then generated *in vitro* using the MEGAshortscript T7 transcription kit (Invitrogen Cat #AM1354) and purified using a MEGAclear transcription clean-up kit (Invitrogen Cat #AM1908) before injection. The donor oligonucleotide for RH mutation was designed with 85 bp 5' homology sequence and 112 bp 3' homology sequence flanking the GA to AC mutation that results in the R1305H amino acid alteration. The single strand donor oligonucleotide was synthesized by IDT technology and PAGE purified. **Genotyping:** For the knock-in model generation, a 574 bp targeted region was amplified from the founder mice using primers outside the homology arms with Phusion Flash PCR master mix (Invitrogen Cat # F548). The primers sequences include GCTGAAGCTGGTGGTAGAGA (forward) and GTGATGCACCCAGCGGTAGT (reverse). This PCR fragment was analyzed by next-generation sequencing (NGS). One founder animal, out of 14 pups born, was identified to have the desired mutation, and it was expanded to establish the R1305H colony. For the knockout model, the targeted region was amplified by PCR (forward primer: CTCATGAAGACCATGGACAACG; reverse primer: CCCAGACCTGCTATCTAGATGTC), generating a 524 bp fragment, and the product analyzed by NGS. One founder mouse, out of 15 pups born, containing a 10 bp deletion (beginning at the codon corresponding to amino acid V756) was chosen to establish the knockout colony. The depletion of the $Ca_V3.3$ protein was confirmed by Western blotting using custom antibody

(immunizing epitope: QHEAGRRPSGLGSTDSGQE; YenZyme antibodies, CA) and by qPCR (forward primer: GAAGCTGGTGCGCTTCAT; reverse primer: TGTCAGGGACTGTGTCTCCA).

**Biochemistry:**

We followed the protocol from Li et al[2] with slight modifications. Briefly, to prepare for crude synaptoneurosomal preparation, cortical tissue from mice 5-7 weeks of age was dissected and homogenized in a solution containing 0.32 M sucrose, 20 mM HEPES (pH 7.4), and protease inhibitor cocktail (Roche # 05 892 791 001). The homogenate was centrifuged for 10 min at 2,800 rpm at 4ºC. The pellet (nuclear fraction) contained nuclei and large cell debris. The supernatant was centrifuged at 14,000 rpm for 10 min. After centrifugation, the supernatant (cytosolic fraction) was removed and the pellet (crude synaptoneurosomal preparation, which is enriched with membrane proteins (Supp Fig 1e) was washed in homogenizing buffer, centrifuged, and solubilized in protein lysis buffer (50 mM Tris-HCl, pH 7.5; 150 mM NaCl, 1% Triton X-100, 0.1% SDS, 1X protease inhibitor cocktail) for 10 min at room temperature. Whole cell lysates were generated by homogenizing cortical tissue with lysis buffer (20 mM Tris.Cl (pH 7.5), 150 mM Tris.Cl, 1% Triton-X100, with fresh protease inhibitor cocktail (Roche # 05 892 791 001) and phosphatase inhibitor (Sigma-Aldrich 4906837001), centrifugation at 14,000 rpm, and supernatants were collected. Whole cell cortical lysates from *Cacna1i*$^{-/-}$ and *Cacna1i*$^{+/+}$ animals serve as negative and positive controls, respectively. Protein concentration was determined by BCA protein assay. For western blotting, equal amount of proteins (30 µg) for each sample were loaded into 3-8% NuPage Tris-Acetate PAGE gel (Invitrogen) for electrophoresis. Proteins were transferred to nitrocellulose membrane using Trans-blot semi-dry apparatus (Bio-Rad # 1704150). The blots were cut at 150 kD, with the upper part for $Ca_V3.3$ detection and the lower part for internal control detection, respectively. The membranes were subsequently blocked with 5% milk in TBST (TBS + 0.1% Tween-20) for 1 hr and kept with primary antibodies overnight at 4

ºC. The following primary antibodies were used: Cav3.3 (custom antibody), vinculin (Cell Signaling Technology # 13091) and Na-K-ATPase (Cell Signaling Technology #3010). The next day, blots were washed three times in TBST and incubated with horseradish peroxidase conjugated anti-rabbit secondary antibody (Santa Cruz # sc-2054) for 1 hr. After final three washes with TBST, bands were detected using SuperSignal West Pico Plus (Thermo Fisher #34580) enhanced chemiluminescence. Densitometric analysis of each protein was conducted using NIH Image J software. Immunoreactivity was normalized to *Cacna1i*$^{+/+}$ control group for each protein. N=6-8 animals/group.

**Slice electrophysiology:**

Whole cell patch-clamp recordings were performed using angled-coronal thalamocortical slices[3] prepared fresh every morning from 21-30 day old male C57BL6 mice. In short, animals were transcardially perfused under isoflurane anesthesia with ice-cold cutting solution (in mM: 194 Sucrose, 30 NaCl, 4.5 KCl, 1.2 $NaH_2PO4$, 10 Glucose, 2 $MgCl_2$, 0.2 $CaCl_2$ and 26 $NaHCO_3$). The animal was then decapitated and the brain carefully dissected out. The cerebellum was removed from the brain and it was placed on a ~10° ramp with the ventral side facing down and the anterior end downhill of the ramp. A vertical cut was then performed at an angle of 55° to the right of the posterior-to-anterior axis of the brain. The tissue anterior to the vertical cut was discarded and the remaining tissue was glued onto the stage of a Leica Vibratome with the cut surface down and the pial surface towards the blade. 250 micron thick slices containing the TRN were then collected and recovered in regular artificial cerebrospinal fluid (aCSF; in mM: 119 NaCl, 2.3 KCl, 1 $NaH_2PO4$; 11 Glucose, 1.3 $MgCl_2$, 2.5 $CaCl_2$, 26 $NaHCO_3$) heated to 32°C for 10 min and then at room temperature aCSF for at least 1 hr. All buffers were continuously bubbled with 95% $O_2$/5% $CO_2$. Subsequently slices were transferred to a 30-32°C submersion recording chamber where they were perfused with aCSF at a rate of 2 ml/min. Borosilicate glass electrodes (3-5 MOhm resistance) were pulled using a Flaming/Brown micropipette puller (Sutter

Instruments) and electrophysiological properties of TRN neurons were recorded under the whole cell configuration using the pClamp software (Molecular Devices). Under current clamp configuration, hyperpolarization induced rebound burst firing and depolarization induced tonic firing were observed using different current injection protocol ranging from -200 pA to +200 pA (Δ 50 mV) using a K-methanesulfonate based internal solution (in mM: 140 $KMeSO_4$; 10 KCl; 10 HEPES; 0.1 EGTA; 4 Mg-ATP; 0.2 Na-GTP; 10 phosphocreatine). Rebound burst firing was observed from different holding membrane potentials ranging from -80 mV to -55 mV. Tonic firing was observed primarily from a holding potential of around -60 mV. Sampled data was analyzed using Clampfit (Molecular Devices). For rebound burst firing properties, hyperpolarization induced bursts were visually identified and counted for each neuron for the different holding membrane potentials and plotted. Bursts were defined as events containing 2 or more action potentials with a maximum of 70 ms interspike interval. The maximum number of bursts ever observed in a neuron irrespective of holding membrane potential and hyperpolarization amplitude was also plotted as the max number of bursts observed. Other properties of the first of such bursts, such as no. of action potentials, threshold of action potentials in the burst, frequency, duration, latency from the end of hyperpolarization pulse and after-hyperpolarization (AHP) observed at the end of the burst were quantified and plotted. In addition to this, frequency and no. of action potentials observed in the second subsequent burst and the inter-burst interval (IBI) were also calculated for neurons displaying more than one burst. For tonic firing, frequency of action potentials was calculated for each depolarizing current amplitude and plotted. The maximum frequency of tonic firing for each neuron and the fast-AHP (fAHP) observed after each single action potential during tonic firing was also calculated and plotted. When a depolarizing pulse displayed burst firing in neurons, the frequency of tonic firing was calculated following the burst, without including the bursting spikes.

For recording T-type $Ca^{2+}$ currents from TRN neurons, slices were transferred to a submersion recording chamber perfused with a modified aCSF (in mM: 120 NaCl, 3 KCl, 20 TEA-

Cl; 5 CsCl, 10 HEPES, 2 $MgCl_2$, 2.5 $CaCl_2$, 10 HEPES) at room temperature containing 0.5 uM tetrodotoxin (TTX). 2-4 MOhm resistance borosilicate pipettes containing a Cs-methanesulfonate based internal solution (in mM: 130 $CsMeSO_4$; 10 TEA-Cl; 5 $MgCl_2$; 10 HEPES; 10 EGTA; 5 Na-ATP) were used to record $Ca^{2+}$ currents under whole cell voltage clamp configuration. To isolate T-type $Ca^{2+}$ currents from the TRN neurons, we utilized a subtraction technique. All neurons were initially held at -60 mV and then a current-voltage relationship (I-V; -90 mV to +20 mV; $\Delta$ 5 mV steps) of the neurons were measured with (-100 mV I-V protocol) and without (-60 mV I-V protocol) a 1s hyperpolarization to -100 mV. The traces obtained from the -60 mV I-V protocol was then subtracted from the -100 mV I-V protocol to isolate the T-type $Ca^{2+}$ currents using Clampfit and the I-V relationship was calculated from the subtracted traces. Peak current densities were calculated by dividing the peak amplitude by the cell capacitance value.

***In vivo* Electrophysiology:**

12- to 19-week-old mice were deeply anesthetized with isoflurane. One intracranial frontal EEG electrode screw (AP Bregma ~+1.5mm, ML Bregma ~+ 1.5mm) and one intracranial parietal EEG electrode screw (AP Bregma ~-1.3mm, ML Bregma ~+ 2.3mm) and a common ground/reference electrode screw above the cerebellum (AP Lambda ~-1, ML Lambda ~0.0) were chronically implanted using a stereotaxic device (David Kopf Instruments). The electromyogram (EMG) electrodes were placed in the nuchal muscle of mice. Electrodes were soldered to EEG/EMG headmount (Pinnacle Technology Inc., part # 8402-SS, KS, USA). Dental acrylic was used to encase the connections. Mice were tethered to a Pinnacle® recording system for >48 hours habituation in recording chambers following one full week of post-operative recovery. EEG/EMG signals were recorded for 60 hours from the onset of the light phase (7am; ZT0) when animals are mostly inactive and spend more time sleeping. All signals were digitized at a sampling rate of 1,000 Hz, filtered (1–100 Hz bandpass for EEG; 10–1 kHz bandpass for EMG), and acquired using Sirenia Acquisition program (Pinnacle Technology). Sleep scoring was performed

manually using 10 second epochs as previously described[46] with Sirenia Sleep software (Pinnacle Technology Inc.).

**Sleep Spindle Analysis:**

For the 61 mice, we extracted 6 hours of EEG, recorded on two channels from ZT0-6 hrs, and combined these data with manual stage assignments of NREM, REM, wake and unscored – per each 10-second epoch. Differences between genotype groups in distributions of stage durations and the number and type of stage transitions were tested using linear regression models. The sleep spindle analysis pipeline is described below and illustrated in supplementary figure 9.

For the EEG signal data, we performed a series of channel-level and epoch-level checks for likely artifact, by calculating the per-epoch root mean square (RMS), three Hjorth parameters[4], and the proportion of clipped points (signal at its maximum or minimum value). Within each mouse and conditional on manually-assigned sleep stage, we then iteratively masked epochs for which any of these five metrics were outliers (+/- 3 standard deviation units) for either channel, performing this procedure twice. We additionally estimated per-epoch mutual information as well as cross-spectra and magnitude-squared coherence between the two EEG channels and the electromyography (EMG) channel, to detect recordings with unusually high cross-talk between the EEG and/or EMG signals. For each mouse, we generated a series of figures for visual review: a) epoch-level sequences of manually assigned stages, masked epochs, signal RMS and sigma power, b) stage-specific power spectra for each channel, c) the distribution of absolute log delta power, and RMS of the EMG channel, to determine the broad validity of manual staging for that animal, d) a scatter plot of both absolute and relative sigma power between the two EEG channels, during manually-scored NR epochs that passed the initial round of filtering, e) epoch-level sequences of mutual information and coherence measures between channels, f) spectrograms of epoch-level power over the whole night, g) raw EEG signals from exemplar epochs based on affinity propagation clustering[5] of within-individual epoch-by-epoch distance

matrices based on permutation distribution clustering of the EEG[6], to point to channels with unusually high rates of artifact otherwise missed by our primary approach. Based on visual review of these figures, we eliminated 5 mice for which neither EEG channel yielded sufficient signal data (3 *Cacna1i*$^{+/+}$, 1 *Cacna1i*$^{RH/RH}$ and 1 *Cacna1i*$^{+/RH}$). In the remaining mice, genotype groups had broadly similar numbers of epochs removed by the above procedure: there were no statistically significant differences for NR epochs, although there were small, nominally significant differences in REM (*Cacna1i*$^{+/RH}$ *and Cacna1i*$^{RH/RH}$ had 7.4% and 8.5% of epochs removed compared to 8.9% in *Cacna1i*$^{+/+}$ (p = 0.02 and 0.04 respectively), whereas in wakefulness, *Cacna1i*$^{+/-}$ had 7.5% of epochs removed compared to 9.7% in *Cacna1i*$^{+/+}$ (p=0.05).

We estimated per-epoch spectral power using the Welch algorithm and fast Fourier transformation applied to 4 second windows, shifted by 2 second increments and tapered with a Tukey window function (taper length 50%). We calculated absolute and relative band power (delta (1-4 Hz), theta (4-8 Hz), alpha (8-12 Hz), sigma (12-15 Hz) and beta (15 to 30 Hz)).
We detected spindles using Morlet wavelet analysis[7], targeting center frequencies of $F_C$ = 9, 11, 13 and 15 Hz (supplementary figure 10), in analyses performed 1) across all epochs, as well as 2) separately within NR, R and W, to allow for stage-specific differences in background sigma activity.

The differences between these two approaches is that different thresholds for spindle detection will be set, as spindles are detected above a multiplicative factor of that individual's median baseline wavelet power value, either based on all epochs (universal baseline), or all epochs of a particular stage (stage-specific). The primary comparisons described here between genotypes, the comparisons focused on differences between stages, or around NREM-REM transitions, were based on spindles detected across all epochs (i.e. using the universal baseline). Nonetheless, all primary results described in the paper are the same, no matter which approach is used.

We used four target frequencies ($F_C$=9, 11, 13, 15 Hz), with each analysis detecting spindles with a peak frequency approximately +/- 1 Hz (see Supp Figure 5 and 6), although strong spindles may be detected at multiple $F_C$ values. For each individual spindle, the actual frequency can be calculated afterwards. That is, "$F_C$=11 Hz spindles", the group of spindles detected when targeting a center frequency of 11 Hz, is likely to encompass a range of spindle frequencies, mostly between 10 and 12 Hz.

Spindles were defined as intervals for which the wavelet power exceeded a threshold of a) 6 times the (stage-specific) median for that mouse for at least 0.3 seconds (the spindle 'core'), and b) 3 times the (stage-specific) median for at least 0.5 seconds (the 'flanking' waxing/waning region), but not more than 3.0 seconds in total (supplementary figure 10). The threshold of 6 was determined by applying Otsu's method for selecting a threshold that maximizes the between-class variance in wavelet power between putative "spindle" and "non-spindle" intervals, with the flanking threshold being 50% of the core threshold (i.e. 3). Spindles within 0.5 seconds of each other were merged, unless the abovementioned 3.0 second criterion would be violated, in which case they were both excluded. We found that defining thresholds as a multiplicative function of the individual's median wavelet power was more robust compared to using the mean, as wavelet power has a very skewed distribution and outliers (i.e. in large part reflecting true spindles) disproportionately influence the mean but not the median.

After detecting spindles, we recorded the count for each epoch, as well as the following summary statistics: density (spindles per minute), count, mean duration, mean amplitude (peak-to-peak, $\mu$V), mean integrated spindle activity (ISA) per spindle ($ISA_S$), per minute ($ISA_M$) and the total ISA ($ISA_T$), mean number of oscillations, and secondary measures of spindle morphology, including mean chirp (intra-spindle change in frequency) and symmetry indices. The ISA is the area under the curve of the normalized wavelet power during spindle events, thereby reflecting both the duration and amplitude of the typical spindle ($ISA_S$), as well as possibly the rate ($ISA_M$)

or absolute number ($ISA_T$) of occurrences. For the purpose of this study we focused on spindle density, duration and $ISA_S$.

For the channels retained in the primary analyses, epochs masked by the initial outlier procedure were excluded from analyses, as were those with an uncertain manual stage assignment. Considering the within-individual distributions of both absolute and relative spectral band power (including slow and gamma bands) for each retained channel, we additionally masked any epoch that was a statistical outlier (+/- 3 SDs) for any measure. On average, ~9% of epochs were masked per individual by this procedure. This approach is likely conservative, but feasible because of the relatively long recordings for each mouse, which means sufficient data are retained for analysis.

Of the 56 remaining mice, there were 29 for which both EEG channels passed the above QC procedure, with the remaining 27 having only a single channel passing (11 parietal and 16 frontal channels). In order to maximize the effective sample size and thus statistical power, we combined derived estimates of key measures (e.g. of spindle density, etc) across channels for the 29 mice with both channels retained, otherwise selecting the value for the remaining QC-positive channel, such that all analyses are based on an initial *N* of 56 (prior to any subsequent measure-specific outlier removal).

Although not a focus of the current work, there are likely detectable topographic differences between frontal and parietal channels. Importantly, in our sample which channels were retained was not associated with genotype ($p=0.24$), based on Fisher's exact test of the table of genotype (5 levels) by channel (4 levels: neither, frontal, parietal, or both). Furthermore, analyzing the 29 mice with two channels passing QC, we did not observe marked differences in the sample averages for key measures between the two EEG channels: e.g. for relative sigma power ($p=0.4$) or $F_C=11$ Hz spindle density ($p=0.42$). In these same mice, key metrics showed moderate to strong intra-individual, cross-channel correlations, e.g. $r=0.55$ ($p=0.00007$) for relative sigma power, and $r=0.58$ ($p=0.000001$) for $F_C=11$ Hz spindle density, supporting the

decision to base our primary analyses on a set of composite measures, to increase power. Nonetheless, secondary analyses performed on only one of the two channels yielded substantively similar results.

Initially mice were assigned to two *Cacna1i*$^{+/+}$ groups, intended for comparison with KO and RH mice respectively. As we did not observe differences between *Cacna1i*$^{+/+}$ groups for the primary outcome measures, we pooled all *Cacna1i*$^{+/+}$ mice into a single group to increase power.

Based on manual staging, we annotated "stable" epochs as those flanked by similarly scored epochs. We also identified all NREM-REM transitions, grouping those epochs into 20-second bins (i.e. pairs of epochs) labeled $E_{-5}$ to $E_{+1}$ as follows: $E_0$ marks the transition period (the two epochs flanking the transition), $E_{-1}$ marks from 30 to 10 seconds prior to the transition, $E_{-2}$ from 50 to 30 seconds, and so on, and $E_{+1}$ marks from 10 to 30 seconds after the NR/R transition.

Primary analyses between genotype groups controlled for age, sex (2 *Cacna1i*$^{+/+}$ mice were female), and the day (relative to the start of data collection for that animal) as well as the date on which EEG data for that animal were extracted for analysis. For all primary comparisons, statistical outliers (+/- 3 SDs) in the dependent variable were removed from analysis. For analyses of epoch-level data, e.g. around NREM/REM transitions, we first created individual-level means and then performed standard linear regression on these measures.

**Statistics, Reagents and animal models:**

Sample sizes were chosen as per literature standard for biochemistry, slice electrophysiology and in vivo EEG experiments. It has been shown previously in the literature that such sample sizes are sufficient for confident statistical conclusions. A small number of mice in the in vivo EEG experiments were excluded based on poor quality recording, as detailed in the manuscript. For the remaining mice, we removed epochs that were statistical outliers as detailed in the sleep spindle analysis section. For slice physiology and in vivo EEG, the data analysis was performed in a blinded fashion. In particular, rebound bursting and calcium current analysis and the EEG manual sleep scoring for the different genotypes were de-identified before analysis. All

Statistical tests used are listed in the appropriate results section. Non-parametric statistical tests were utilized whenever necessary. There is no estimate of variation and the variance between groups were not statistically compared.

We used a custom-made $Ca_V3.3$ antibody that can be available from us when requested. We validated the $Ca_V3.3$ antibody in the *Cacna1i*$^{-/-}$ animals. The data is provided in Figure 1.

All of the experiments with animals were approved by the Broad Institute IACUC (Institutional Animal Care and Use Committee). All animals used were male C57BL6 mice. We used 0-8 day old mice for biochemistry experiments, 21-30 day old mice for brain slice experiments, whereas, 12-19 week old animals for in vivo EEG experiments. Moreover, for the in vivo EEG experiments and brain slice experiments, mice of different genotype were randomized to testing day/time.

Code availability: The software used for sleep EEG analysis is LUNA developed by SMP (version 0.2, http://zzz.bwh.harvard.edu/luna/, which links to a BitBucket open-source repositry).

**REFERENCES:**


1. Ran FA *et al*. Genome engineering using the CRISPR-Cas9 system. *Nat Protoc* 2013; **8**(11)**:** 2281-2308.

2. Li N *et al.* mTOR-dependent synapse formation underlies the rapid antidepressant effects of NMDA antagonists. *Science* 2010; **329**(5994)**:** 959-964.

3. Agmon A, Connors BW. Thalamocortical responses of mouse somatosensory (barrel) cortex in vitro. *Neuroscience* 1991; **41**(2-3)**:** 365-379.

4. Hjorth B. EEG analysis based on time domain properties. *Electroencephalogr Clin Neurophysiol* 1970; **29**(3)**:** 306-310.

5. Frey BJ, Dueck D. Clustering by passing messages between data points. *Science* 2007; **315**(5814)**:** 972-976.

6. Brandmaier AM. pdc: An R Package for Complexity-Based Clustering of Time Series. *J Stat Softw* 2015; **67**(5)**:** 1-23.

7. Purcell SM *et al.* Characterizing sleep spindles in 11,630 individuals from the National Sleep Research Resource. *Nat Commun* 2017; **8:** 15930.

**Supplementary Figure 1: Human and mouse gene alignment; $Ca_V3.3$ expression in knock-out heterozygous mice and validation of synaptoneurosomal preparations.**

**a.** Alignment of human *CACNA1I* and murine *Cacna1i*. **b.** A typical example of a western blot showing reduction of $Ca_V3.3$ expression in *Cacna1i*$^{+/-}$ cortex and hippocamapal lysates in two different animals (+/-) when compared to wildtype littermates (+/+). *Cacna1i*$^{-/-}$ (-/-) served as the control showing a near complete lack of protein. **c and d.** Quantification from qPCR (n = 5 *Cacna1i*$^{+/+}$; n = 4 *Cacna1i*$^{+/-}$) and western blot experiments from cortical and hippocampal lysates (n = 7 *Cacna1i*$^{+/+}$; n = 7 *Cacna1i*$^{+/-}$) showing approximately 50% reduction in mRNA (c) and protein (d) in the heterozygous knock-out mice (+/-). **e.** Validation for synaptosneurosomal preparations from wildtype cortical lysates showing enrichment of synaptic markers like PSD95 in the synaptoneurosomal lysates (Syn) compared to the cytosolic fraction (Cyto), whereas Lamin B is enriched in the nuclear fraction (Pellet). Whole brain lysates (Whole Lysate) from wildtype (+/+) and knock-out (-/-) served as control. * represents $p < 0.05$; Error bars represent S.E.M.

**Supplementary Figure 2: Tonic firing is unchanged in *Cacna1i*$^{RH/RH}$ and *Cacna1i*$^{-/-}$ mice.**

**a.** Sample traces from wildtype (+/+), *Cacna1i*$^{RH/RH}$ (RH/RH) and *Cacna1i*$^{-/-}$ (-/-) mice showing depolarization induced tonic firing in a representative TRN neuron from a holding potential of -60 mV. **b.** Relationship of tonic firing frequency with different levels of current injections showing similar depolarization induced excitability among wildtype, *Cacna1i*$^{RH/RH}$ and *Cacna1i*$^{-/-}$ TRN neurons. **c and d.** Average of the maximum tonic firing frequency (c) and fast afterhyperpolarization (fAHP) of action potentials (d) observed across all neurons and all genotypes showing no significant differences among them. In each violin plot for c, and d black squares represent individual data points and white boxes represent mean value; length of black line represents the interquartile range.

**Supplementary Figure 3: Relative sigma power in heterozygous *Cacna1i*$^{+/RH}$ and *Cacna1i*$^{+/-}$ mice.**

**a.** Relative sigma power calculated during NREM sleep and plotted normalized to mean wildtype levels showing no differences in the heterozygous genotypes. **b.** Relative sigma power calculated for wildtype and heterozygous *Cacna1i*$^{+/RH}$ and *Cacna1i*$^{+/-}$ mice during NREM epochs (20 s long) preceding a REM episode. No differences were observed in the *Cacna1i*$^{+/RH}$ and *Cacna1i*$^{+/-}$ mice in any of the epochs including the NREM epoch immediately prior to a REM episode, where all three genotypes show an increase in relative sigma power. ** represents $p < 0.01$; in the violin plot black squares represent individual data points and white boxes represent mean value; length of black line represents the interquartile range.

**Supplementary Figure 4: Relative theta power in all genotypes.**

Relative theta power calculated during NREM sleep and plotted normalized to mean wildtype levels showing a significant increase in *Cacna1i*$^{-/-}$ and *Cacna1i*$^{+/-}$ mice with no difference in the *Cacna1i*$^{RH/RH}$ or *Cacna1i*$^{+/RH}$ genotypes. * denotes $p<0.05$ and ** denotes $p<0.01$.

**Supplementary Figure 5: NREM Spindle density for all genotypes and all center frequencies.**

**a-c.** Spindle density distribution for $F_C$=9 Hz (a), 11 Hz (b), 13 Hz (c) and 15 Hz (d) normalized to average wildtype levels, in *Cacna1i*$^{+/+}$, *Cacna1i*$^{RH/RH}$, *Cacna1i*$^{-/-}$, *Cacna1i*$^{+/RH}$ and *Cacna1i*$^{+/-}$ animals showing significant reductions in *Cacna1i*$^{RH/RH}$ and *Cacna1i*$^{-/-}$ mice for 13 and 15 Hz spindles but not in 9 Hz spindle density. No changes were observed for any center frequencies in *Cacna1i*$^{+/RH}$ and *Cacna1i*$^{+/-}$ animals. * represents $p < 0.05$; ** represents $p < 0.01$; in the violin plots black squares represent individual data points and white boxes represent mean value; length of black line represents the interquartile range.

**Supplementary Figure 6: Spindle density in NREM, REM and Wake for heterozygous *Cacna1i*$^{+/RH}$ and *Cacna1i*$^{+/-}$ mice for spindles with 9, 11, 13 and 15 Hz center frequency.**

**a.** NREM, REM and wake average spindle density quantified as spindles per minute for $F_C$=9,11,13 and 15 Hz spindles across wildtype, heterozygous *Cacna1i*$^{+/RH}$ and heterozygous *Cacna1i*$^{+/-}$ animals showing no significant differences in sleep spindles during any of the sleep stages in both *Cacna1i*$^{+/RH}$ and *Cacna1i*$^{+/-}$ animals compared to wildtype *Cacna1i*$^{+/+}$.

**Supplementary Figure 7: Spindle density for heterozygous *Cacna1i*$^{+/RH}$ and *Cacna1i*$^{+/-}$ mice during NREM-REM transitions.**

**a.-d.** Average spindle density of $F_C$=9 Hz (a); 11Hz (b); 13 Hz (c); 15 Hz (d) spindles for NREM epochs (each 20 seconds) preceding a REM episode, showing an increase in mean spindle density during NREM-REM transition (dashed vertical line). No significant changes of spindle density are observed in both *Cacna1i*$^{+/RH}$ and *Cacna1i*$^{+/-}$ animals during the transition compared to the wildtype. # ($p < 0.05$) and ## ($p < 0.01$) denotes statistical significance between *Cacna1i*$^{+/+}$ and *Cacna1i*$^{RH/RH}$. * ($p < 0.05$),** ($p < 0.01$) and *** ($p < 0.001$) denotes statistical significance between *Cacna1i*$^{+/+}$ and *Cacna1i*$^{-/-}$ animals. Error bar represents S.E.M.

**Supplementary Figure 8: NREM Spindle characteristics for heterozygous *Cacna1i*$^{+/RH}$ and *Cacna1i*$^{+/-}$ mice for spindles with 11 Hz center frequency during.**

**a and b,** Characteristics of $F_C$=11 Hz spindles including integrated spindle activity per spindle ($ISA_s$; a) and duration (b), normalized to *Cacna1i*$^{+/+}$ average values showing no significant changes in the distribution of spindle duration and $ISA_s$ in the *Cacna1i*$^{+/RH}$ and *Cacna1i*$^{+/-}$ animals as opposed to homozygous *Cacna1i*$^{RH/RH}$ and *Cacna1i*$^{-/-}$ mice who have significant reductions in both spindle duration and $ISA_s$ when compared to *Cacna1i*$^{+/+}$ animals. ** represents $p < 0.01$; *** represents $p < 0.001$; in the violin plots black squares represent

individual data points and white boxes represent mean value; length of black line represents the interquartile range.

**Supplementary Figure 9: Schematic of quality control (QC) and analysis steps. All figures show real data for a single WT mouse as an example.**

**a.** Epoch-level statistics (e.g. signal root mean square (RMS) and spectral band power) to identifying statistical outliers. **b.** Permutation distribution distance analysis of raw EEG epochs followed by affinity propagation clustering to facilitate visual review of signals. **c.** Manual inspection of epoch-level spectrograms. **d.** Power spectral densities for NR, R and W sleep stages. **e.** Sigma power calculated around NR/R transitions. **f.** Manually staged 10-second epochs (NR blue, R orange, W gray, black lines at bottom show epochs masked during QC). **g.** Automatically detected spindles, at four different target frequencies, showing clear enrichment during NR sleep. **h.** Single 10-second epoch during NR sleep, given a manually detected ~13 Hz spindle (green bar) and raw EEG trace below.

**Supplementary Figure 10: Schematic of wavelet-based spindle detection.**

Schematic showing example of a detected spindle and the parameters and steps involved in our wavelet-based spindle detection. CWT: continuous wavelet transform.

## Supplementary Figure 1

**a.**

```
Hu_CACNA1I  1340 TRNITNRSDC 1349
Ms_Cacna1i  1299 TRNITNRSDC 1308
```

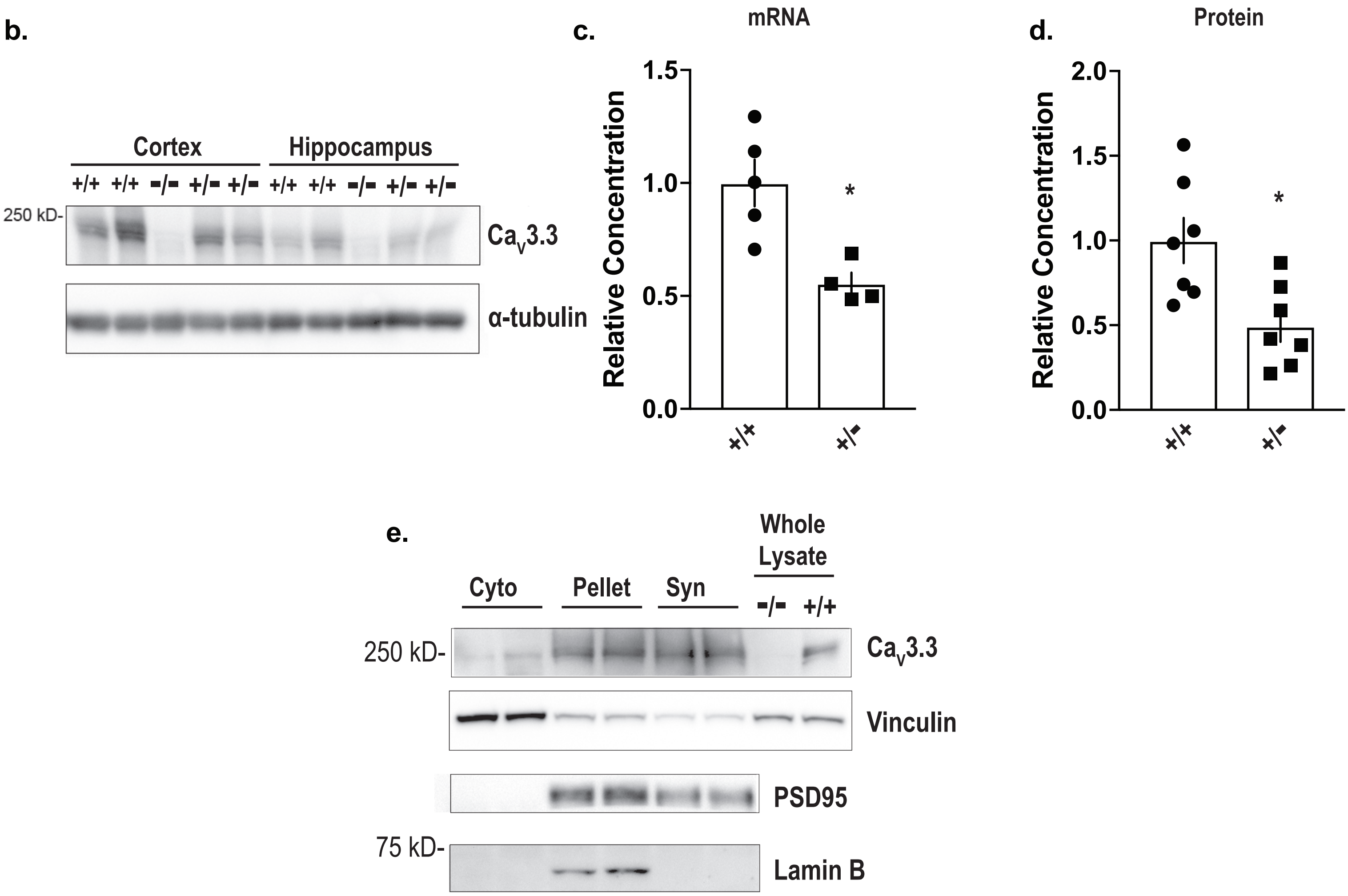

Supplementary Figure 2

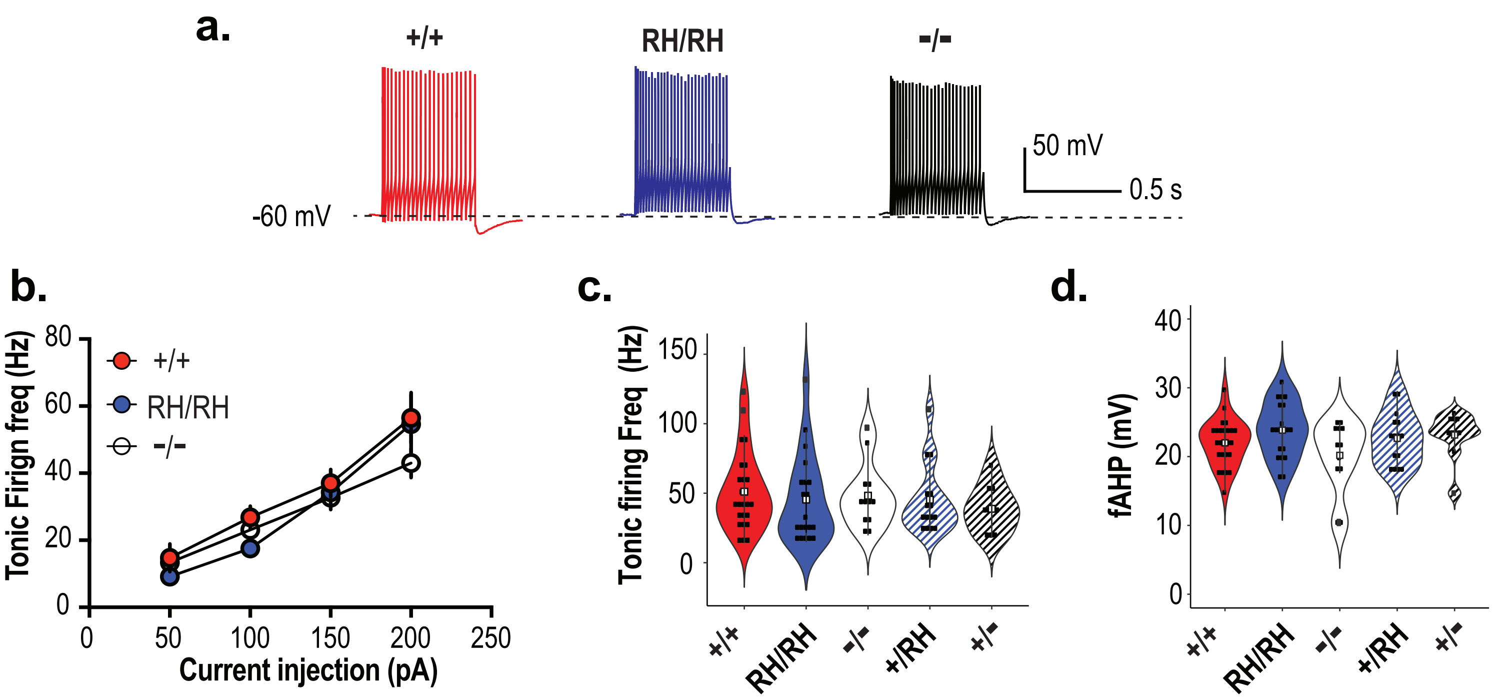

## Supplementary Figure 3

**a.**

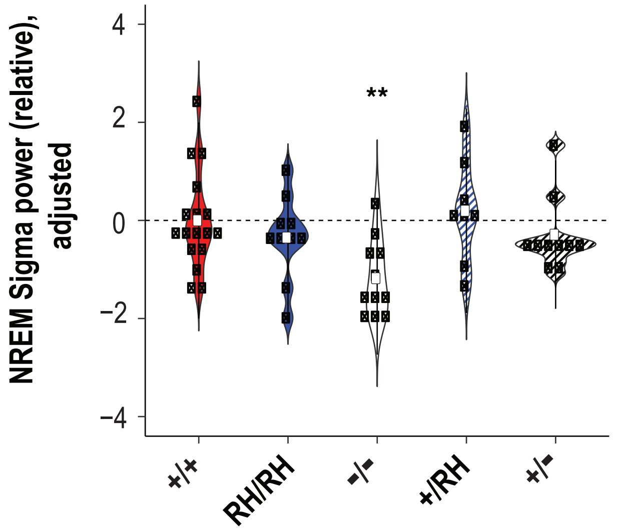


**b.**

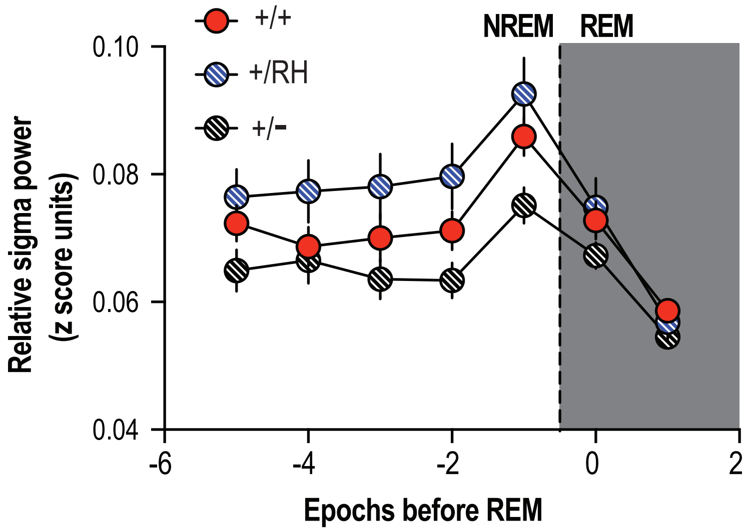

**Supplementary Figure 4**

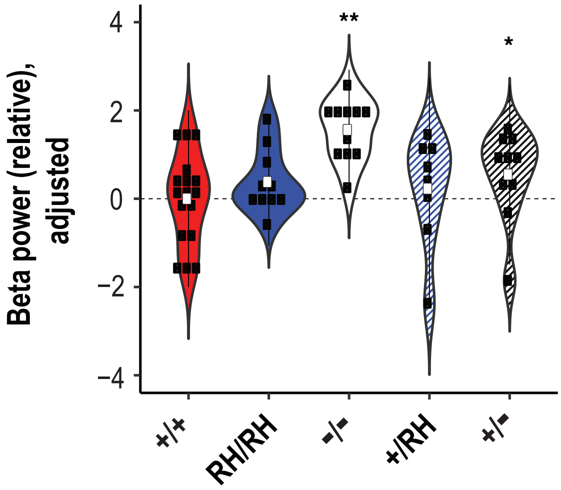

**Supplementary Figure 5**

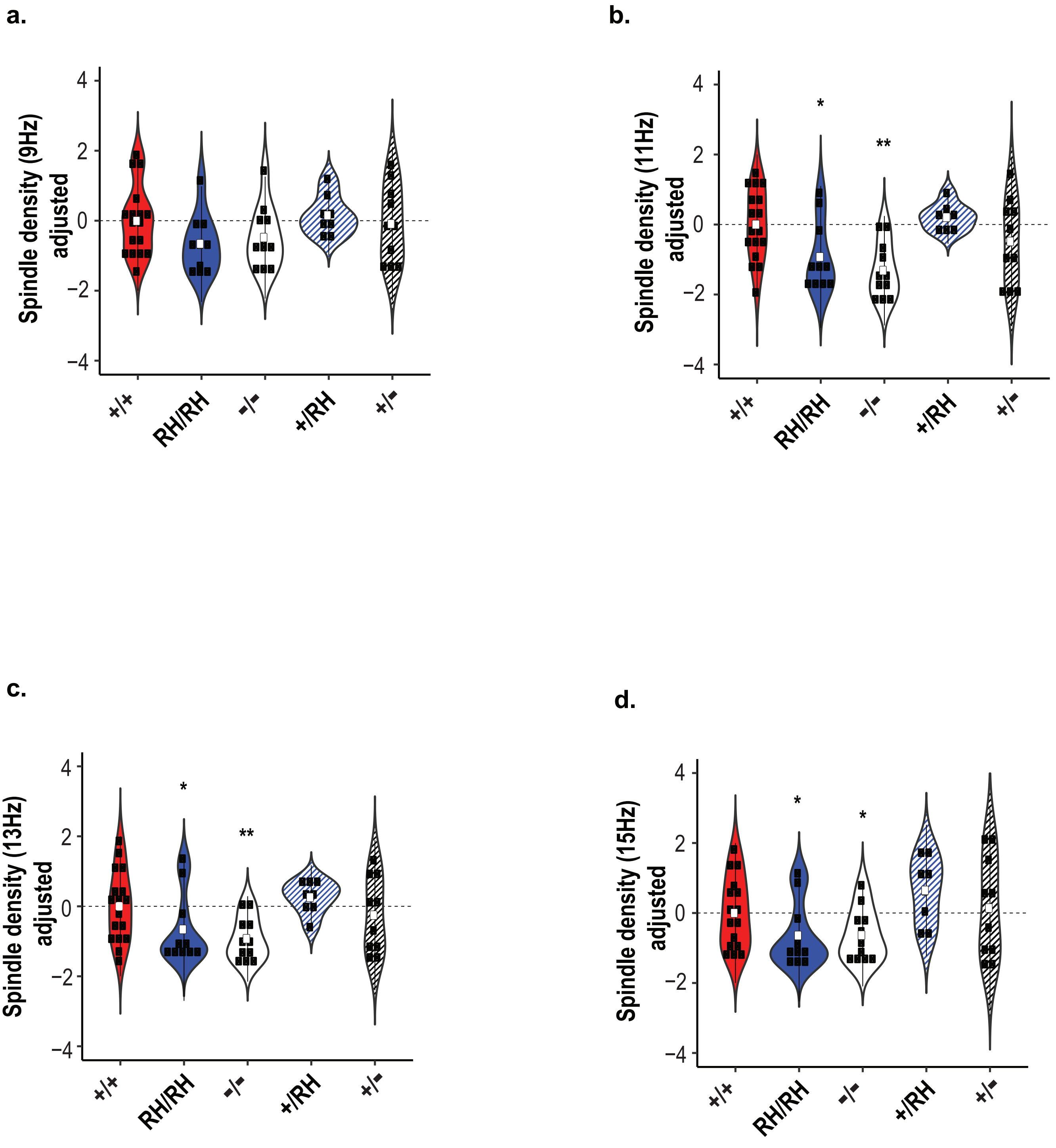

## Supplementary Figure 6

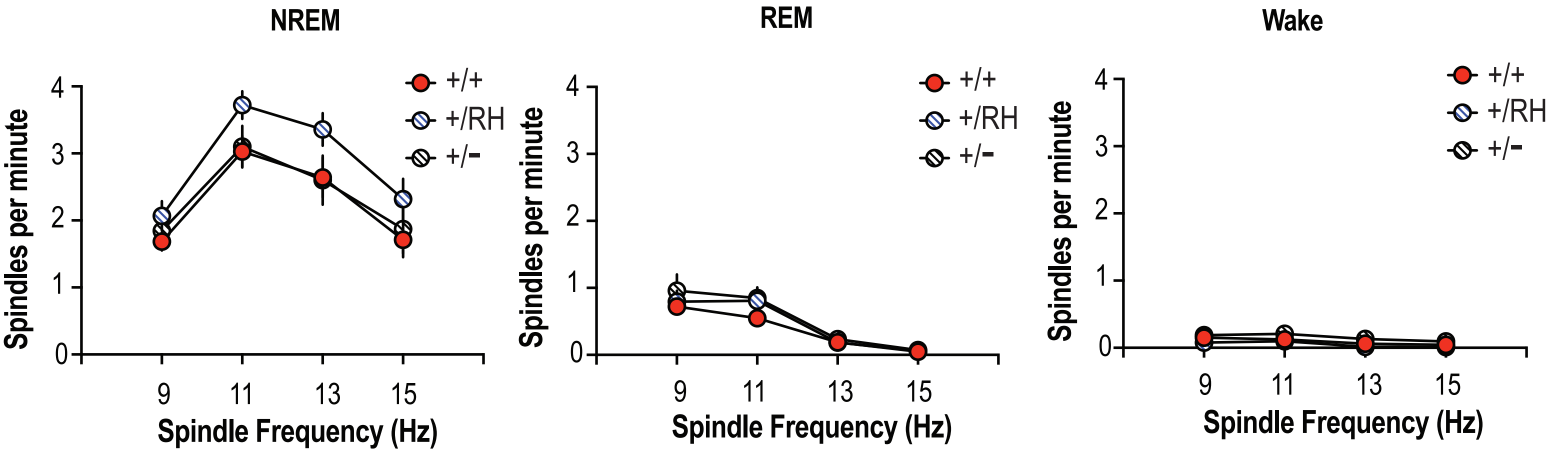

## Supplementary Figure 7

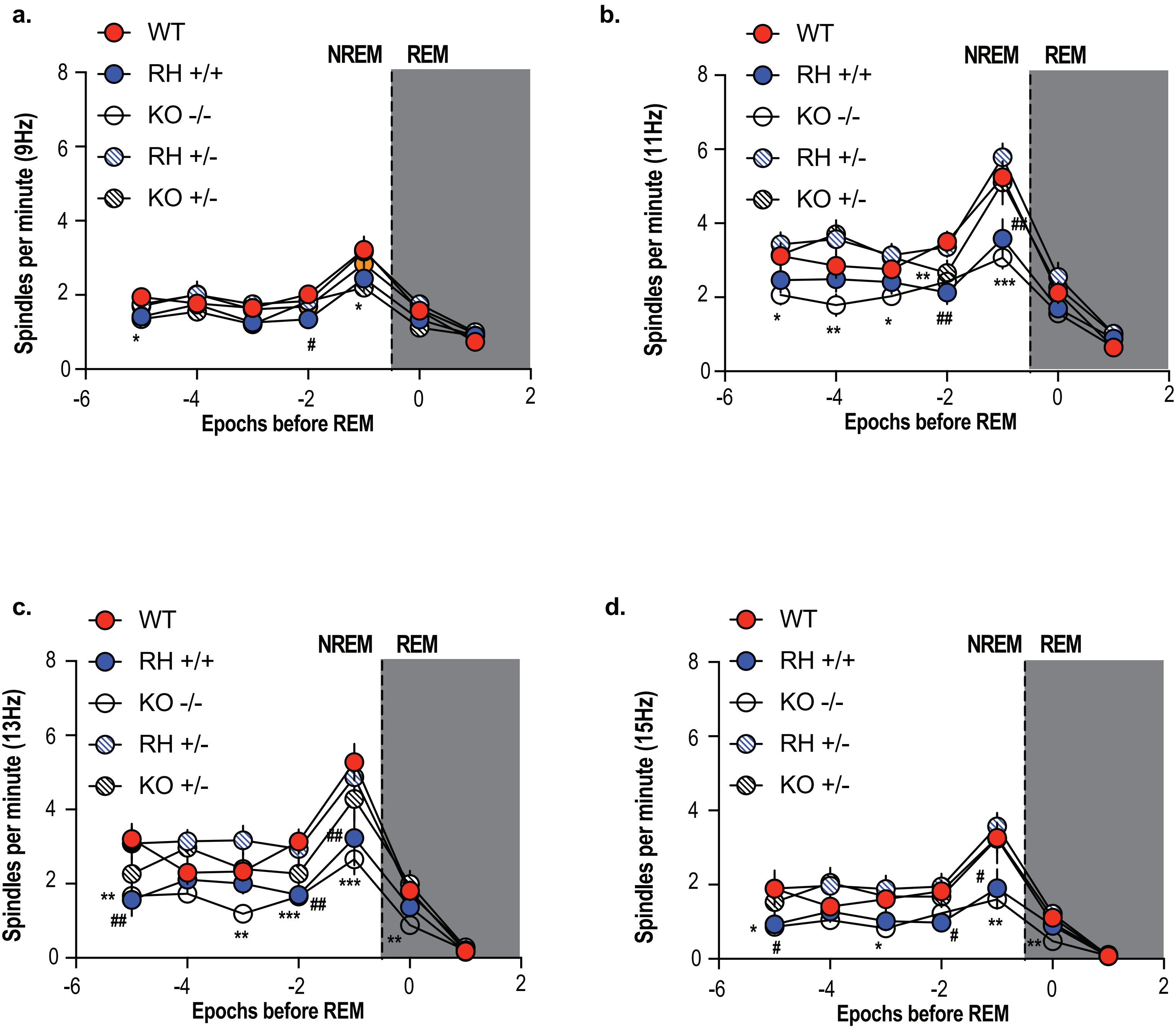

**Supplementary Figure 8**

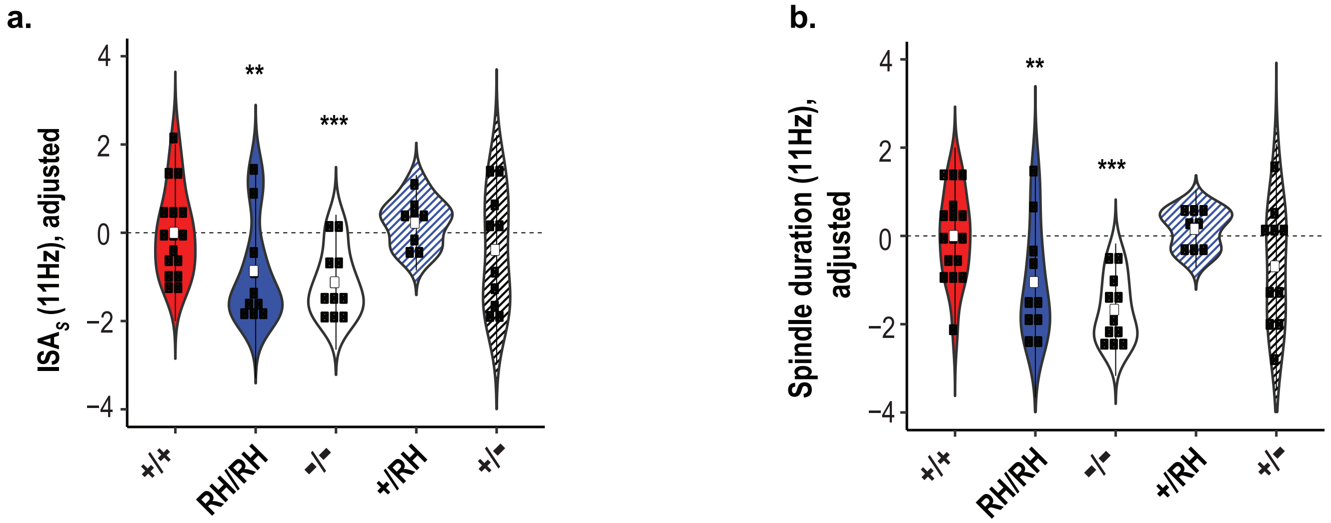

# Supplementary Figure 9

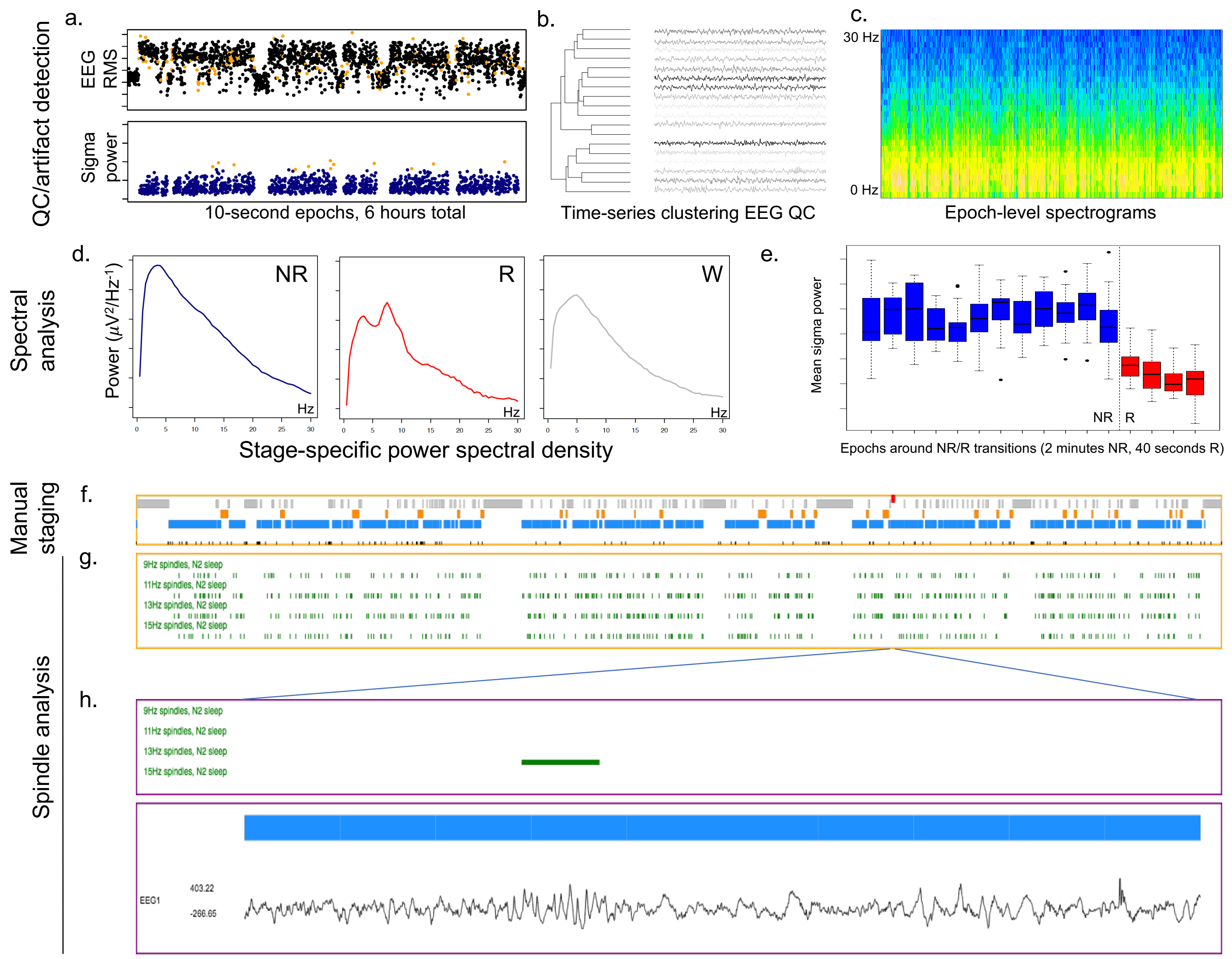

## Supplementary Figure 10

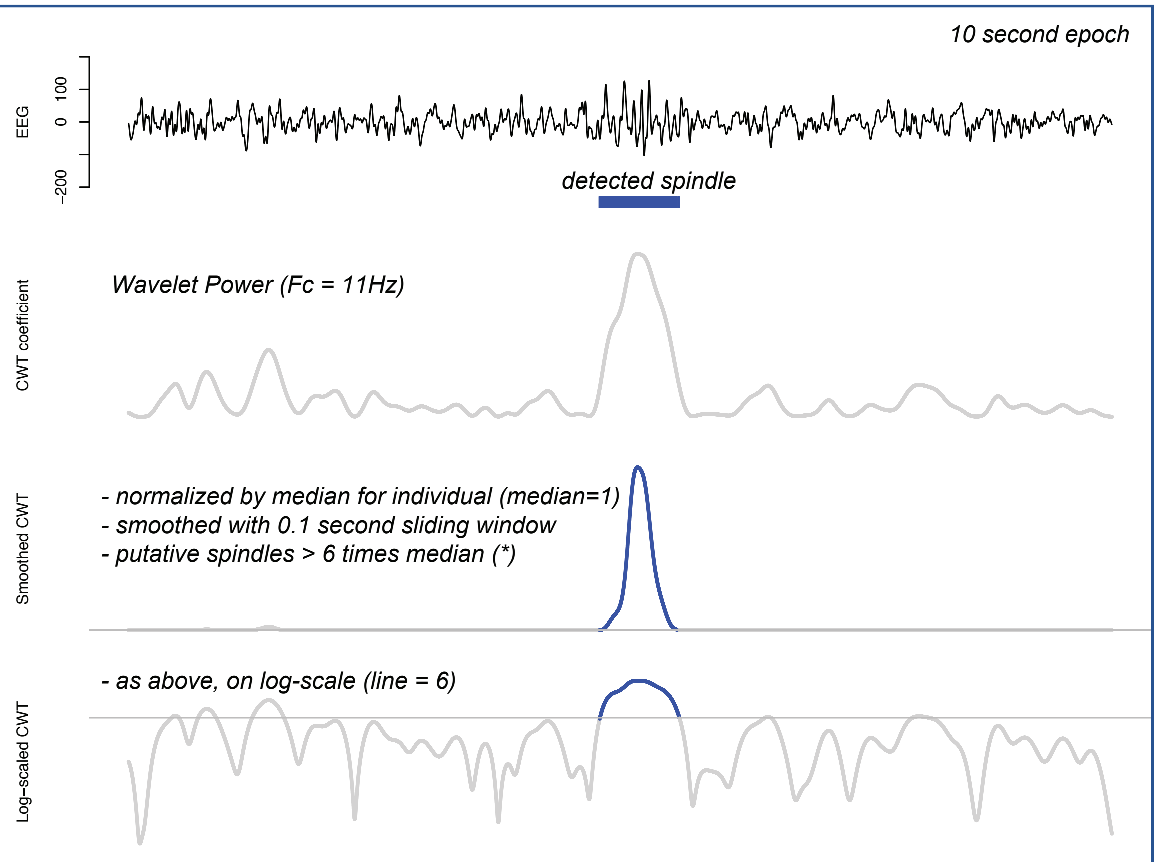


*Spindle thresholds:* *- above 6 times median for 0.3 seconds and above 3 times median for 0.5 seconds*
*- spindles within 0.5 seconds merged*
*- maximum spindle duration of 3.0 seconds*

*Spindle parameters:* *count, density (count per minute), frequency, duration, # oscillations, amplitude, integrated spindle activity (area under blue curve), chirp and symmetry indices*

| | *Cacna1i*$^{+/+}$ | *Cacna1i*$^{RH/RH}$ | *Cacna1i*$^{-/-}$ | *Cacna1i*$^{+/RH}$ | *Cacna1i*$^{+/-}$ | ANOVA |
|---|---|---|---|---|---|---|
| **Mean peak T-type Current density (cohort 1) (pA/pF)** | -7.6 ± 0.8; (N=12) | -4.9 ± 0.5 (N=12; p=0.0101) | -2.1 ± 0.5; (N=11; p<0.0001) | NA | NA | p<0.0001; $R^2$ = 0.563; F (2,32) = 2.181 |
| **Mean peak T-type Current density (cohort 2) (pA/pF)** | -6.1 ± 0.6; (N=10) | NA | NA | -6.8 ± 0.6; (N=7; p=0.53) | -3.9 ± 0.4; (N=10; p=0.0135) | p=0.0027; $R^2$ = 0.39; F (2,24) = 1.882 |
| **Mean peak HVA Current density (cohort 1) (pA/pF)** | -8.0 ± 1.1; (N=12) | -7.1 ± 0.9; (N=14) | -7.5 ± 1.1; (N=11) | NA | NA | p=0.80; $R^2$ = 0.013; F (2,34) = 0.1574 |
| **Mean peak HVA Current density (cohort 2) (pA/pF)** | -6.1 ± 0.6; (N=10) | NA | NA | -7.9 ± 1.5; (N=7) | -8.1 ± 1.1; (N=12) | p=0.58; $R^2$ = 0.0448; F (2,24) = 0.7681 |
| **Avg. of Max number of Bursts** | 2.6 ± 0.18; (N=26) | 1.4 ± 0.09; (N=30; p=0.0001) | 0.4 ± 0.15; (N=12; p=0.0001) | 2.4 ± 0.45; (N=15; p=0.8988) | 1.9 ± 0.32; (N=10; p=0.1552) | p < 0.0001; $R^2$ = 0.3836; F (4,88) = 13.69 |
| **Avg. First Burst Frequency (Hz)** | 203.6 ± 12.66; (N =26) | 144.8 ± 10.38; (N=29; p=0.0009) | 22.9 ± 7.38; (N = 5; p = 0.0001) | 208.0 ± 15.03; (N=13; p=0.9979) | 175.8 ± 14.69; (N=10; p=0.4843) | p < 0.0001; $R^2$ = 0.4143; F (4,78) = 13.79 |
| **Avg. Number of AP in First Burst** | 4.9 ± 0.24; (N =26) | 3.8 ± 0.21; (N=29; p=0.0038) | 2.2 ± 0.20; (N=5; p= 0.0001) | 4.6 ± 0.31; (N=13; p=0.8610) | 4.6 ± 0.48; (N=10; p=0.8774) | p < 0.0001; $R^2$ = 0.2701; F (4,78) = 7.22; |
| **Avg. peak AHP (mV)** | 26.09 ± 1.38; (N =26) | 19.09 ± 1.37; (N=29; p=0.0012) | 3.09 ± 1.02; (N=5; p = 0.0001) | 30.64 ± 1.59; (N=13; p=0.1671) | 21.49 ± 2.34; (N=10; p=0.2243) | p < 0.0001; $R^2$ = 0.4824; F (4,78) = 18.17 |
| **Avg. Latency (ms)** | 66.61 ± 6.83; (N=26) | 60.35 ± 4.27; (N=29) | 56.04 ± 4.30; (N=5) | 60.84 ± 5.59; (N=13) | 68.65 ± 7.82; (N=10) | p = 0.7819; $R^2$ = 0.0219; F (4,78) = 0.4365 |
| **Avg. Duration (ms)** | 20.66 ± 1.56; (N=26) | 22.39 ± 3.17; (N=29; p=0.9597) | 62.94 ± 8.91; (N=5; p=0.0001) | 17.59 ± 1.34; (N=13; p=0.8765) | 21.19 ± 1.72; (N=10; p=0.8765) | p < 0.0001; $R^2$ = 0.4179; F (4,78) = 14.0 |
| **Avg. Second Burst Frequency (Hz)** | 119.4 ± 11.3; (N=23) | 52.22 ± 5.98; (N=12; p=0.0002) | NA | 135.0 ± 12.34; (N=11; p=0.6329) | 87.32 ± 9.45; (N=6; p=0.2533) | p < 0.0001; $R^2$ = 0.361; F (3,48) = 9.04 |
| **Avg. Number of AP in Second Burst** | 3.74 ± 0.25; (N=23) | 2.25 ± 0.13; (N=12; p=0.0021) | NA | 4.55 ± 0.45; (N=11; p=0.1566) | 4.33 ± 0.49; (N=6; p=0.5483) | p = 0.0001; $R^2$ = 0.3529; F (3,48) = 8.73; |
| **Avg. IBI (ms)** | 206.2 ± 16.79; (N=23) | 198.9 ± 23.55; (N=12) | NA | 210.5 ± 18.48; (N=11) | 239.4 ± 28.39; (N=6) | p = 0.4027; $R^2$ = 0.0246; F (3,48) = 0.4027 |
| **Avg. Threshold of AP (mV)** | -44.63 ± 0.72; (N=26) | -43.46 ± 0.87; (N=29) | -42.17 ± 1.38; (N=5) | -44.51 ± 1.37; (N=13) | -44.57 ± 1.11; (N=10) | p = 0.6717; $R^2$ = 0.0293; F (4,78) = 0.5888 |
| **Avg. Tonic Firing Frequency (Hz)** | 51.12 ± 5.61; (N = 25) | 45.41 ± 7.23; (N = 20) | 48.24 ± 6.75; (N = 12) | 45.33 ± 6.46; (N = 15) | 38.80 ± 5.23; (N = 10) | p = 0.7909; $R^2$ = 0.0216; F (4,77) = 0.4239; |

| | | | | | | |
|---|---|---|---|---|---|---|
| **Avg. fAHP (mV)** | 22.00 ± 0.67; (N = 25) | 23.84 ± 0.89; (N = 20) | 20.18 ± 1.49; (N = 12) | 22.65 ± 0.97; (N = 15) | 23.14 ± 1.07; (N = 10) | p = 0.1306; $R^2$ = 0.0871; F (4,77) = 1.835 |
| **Input Resistance (mOhm)** | 194.2 ± 8.14; (N=23) | 209.7 ± 11.59; (N=21) | 209.1 ± 10.06; (N=12) | 184.9 ± 14.43; (N=15) | 187.8 ± 9.63; (N=10) | p = 0.4058; $R^2$ = 0.0506; F (4,58) = 1.014 |
| **Resting membrane potential (mV)** | -65.84 ± 1.52; (N=26) | -65.81 ± 1.39; (N=29) | -63.99 ± 3.01; (N=8) | -69.46 ± 2.20; (N=15) | -72.85 ± 1.60; (N=10) | p = 0.0501; $R^2$ = 0.1056; F (4,84) = 2.479 |

**Supplementary Table 1: LVA and HVA current density, rebound burst parameters, tonic firing parameters and passive membrane properties of TRN neurons of all genotypes.** Mean ± SEM and the number of cells recorded (N) are listed for each genotype. One-way ANOVA test results as well as multiple comparison test p values are reported. Colored cells indicate statistically significant differences in the overall one-way ANOVA test or when compared to the control group wildtype (*Cacna1i*$^{+/+}$) using Dunnett’s multiple comparison test.

| | ***Cacna1i***$^{+/+}$ (N=17) | ***Cacna1i***$^{RH/RH}$ (N=10) | ***Cacna1i***$^{-/-}$ (N=5) | ***Cacna1i***$^{+/RH}$ (N=13) | ***Cacna1i***$^{+/-}$ (N=10) |
|---|---|---|---|---|---|
| **All Transitions** | 0.11 ± 0.009; | 0.10 ± 0.009; (p=0.725) | 0.11 ± 0.012; (p=0.557) | 0.08 ± 0.007; (p=0.118) | 0.11 ± 0.009; (p=0.525) |
| **From NREM** | 0.09 ± 0.012; | 0.08 ± 0.013; (p=0.411) | 0.09 ± 0.012; (p=0.973) | 0.07 ± 0.011; (p=0.323) | 0.10 ± 0.009; (p=0.466) |
| **From REM** | 0.17 ± 0.007; | 0.15 ± 0.006; (p=0.205) | 0.17 ± 0.015; (p=0.643) | 0.16 ± 0.007; (p=0.695) | 0.16 ± 0.009; (p=0.960) |
| **From Wake** | 0.13 ± 0.009; | 0.14 ± 0.013; (p=0.260) | 0.15 ± 0.009; (p=0.166) | 0.11 ± 0.007; (p=0.035) | 0.13 ± 0.016; (p=0.923) |

**Supplementary Table 2: Sleep transitions across genotypes.** Mean ± SEM of probability of sleep transitions from different sleep stages are listed. N represents number of animals analyzed for each genotype. p values are also reported for each genotype when compared to wildtype (*Cacna1i*$^{+/+}$), showing most genotypes with no significant differences in sleep transitions when compared to *Cacna1i*$^{+/+}$. Colored cells indicate statistically significant differences when compared to the control group *Cacna1i*$^{+/+}$.

| | *Cacna1i*$^{+/+}$ | *Cacna1i*$^{RH/RH}$ | *Cacan1i*$^{-/-}$ | *Cacna1i*$^{+/RH}$ | *Cacna1i*$^{+/-}$ |
|---|---|---|---|---|---|
| **NREM 9 Hz Avg. Spindle Density (spindles/min)** | 1.69 ± 0.13; (N=17) | 1.38 ± 0.15; (N=10; p=0.1405) | 1.59 ± 0.12; (N=11; p=0.1684) | 2.07 ± 0.22; (N=8; p=0.8154) | 1.84 ± 0.24; (N=10; p=0.9556) |
| **NREM 11 Hz Avg. Spindle Density (spindles/min)** | 3.02 ± 0.24; (N=17) | 2.36 ± 0.26; (N=10; p=0.0271) | 2.12 ± 0.17; (N=11; p=0.0018) | 3.72 ± 0.21; (N=8; p=0.5045) | 3.10 ± 0.31; (N=10; p=0.7939) |
| **NREM 13 Hz Avg. Spindle Density (spindles/min)** | 2.64 ± 0.30; (N=17) | 1.80 ± 0.31; (N=10; p=0.0246) | 1.56 ± 0.17; (N=11; p=0.0017) | 3.36 ± 0.25; (N=8; p=0.7230) | 2.60 ± 0.37; (N=10; p=0.4810) |
| **NREM 15 Hz Avg. Spindle Density (spindles/min)** | 1.71 ± 0.26; (N=17) | 0.98 ± 0.21; (N=10; p=0.0282) | 1.04 ± 0.15; (N=11; p=0.0111) | 2.32 ± 0.30; (N=8; p=0.8689) | 1.87 ± 0.37; (N=10; p=0.8624) |
| **REM 9 Hz Avg. Spindle Density (spindles/min)** | 0.72 ± 0.09; (N=17) | 0.85 ± 0.23; (N=10; p=0.5055) | 0.87 ± 0.20; (N=11; p=0.6837) | 0.79 ± 0.17; (N=8; p=0.7177) | 0.96 ± 0.25; (N=9; p=0.4177) |
| **REM 11 Hz Avg. Spindle Density (spindles/min)** | 0.55 ± 0.07; (N=17) | 0.75 ± 0.17; (N=10; p=0.3374) | 0.75 ± 0.21; (N=11; p=0.2921) | 0.81 ± 0.20; (N=8; p=0.2693) | 0.84 ± 0.16; (N=9; p=0.1769) |
| **REM 13 Hz Avg. Spindle Density (spindles/min)** | 0.19 ± 0.05; (N=17) | 0.10 ± 0.03; (N=10; p=0.4680) | 0.18 ± 0.06; (N=10; p=0.9906) | 0.19 ± 0.06; (N=8; p=0.9738) | 0.24 ± 0.06; (N=10; p=0.8765) |
| **REM 15 Hz Avg. Spindle Density (spindles/min)** | 0.05 ± 0.02; (N=16) | 0.06 ± 0.02; (N=10; p=0.8112) | 0.04 ± 0.02; (N=10; p=0.7038) | 0.06 ± 0.02; (N=8; p=0.6956) | 0.07 ± 0.03; (N=10; p=0.5998) |
| **Wake 9 Hz Avg. Spindle Density (spindles/min)** | 0.15 ± 0.04; (N=17) | 0.19 ± 0.03; (N=10; p=0.3470) | 0.11 ± 0.02; (N=10; p=0.6917) | 0.08 ± 0.03; (N=7; p=0.4950) | 0.19 ± 0.04; (N=10; p=0.3794) |
| **Wake 11 Hz Avg. Spindle Density (spindles/min)** | 0.12 ± 0.02; (N=16) | 0.18 ± 0.04; (N=10; p=0.2385) | 0.11 ± 0.03; (N=10; p=0.8342) | 0.10 ± 0.03; (N=7; p=0.6738) | 0.21 ± 0.06; (N=10; p=0.1694) |
| **Wake 13 Hz Avg. Spindle Density (spindles/min)** | 0.06 ± 0.01; (N=16) | 0.06 ± 0.02; (N=10; p=0.9808) | 0.13 ± 0.07; (N=11; p=0.1045) | 0.02 ± 0.00; (N=7; p=0.4360) | 0.13 ± 0.06; (N=10; p=0.2692) |
| **Wake 15 Hz Avg. Spindle Density (spindles/min)** | 0.05 ± 0.01; (N=16) | 0.07 ± 0.03; (N=10; p=0.2398) | 0.03 ± 0.02; (N=10; p=0.4109) | 0.01 ± 0.00; (N=7; p=0.6719) | 0.10 ± 0.04; (N=10; p=0.2141) |

**Supplementary Table 3: NREM, REM and Wake sleep spindle density of all genotypes.** Mean ± SEM and the number of animals recorded (N) are listed for each genotype. p values reported reflect statistical comparison with the WT group. Colored cells indicated statistically significant differences when compared to the control group *Cacna1I*$^{+/+}$. Significant changes were observed during NREM without affecting spindle density during REM and wake.

| | *Cacna1i*$^{+/+}$ | *Cacna1i*$^{RH/RH}$ | *Cacna1i*$^{-/-}$ | *Cacna1i*$^{+/RH}$ | *Cacna1i*$^{+/-}$ |
|---|---|---|---|---|---|
| **9 Hz Avg. NREM Spindle Duration (ms)** | 0.78 ± 0.02; (N=16) | 0.74 ± 0.01; (N=10; p=0.0041) | 0.75 ± 0.01; (N=11; p=0.0297) | 0.79 ± 0.01; (N=8; p=0.1467) | 0.77 ± 0.03; (N=10; p=0.4375) |
| **11 Hz Avg. NREM Spindle Duration (ms)** | 0.82 ± 0.02; (N=16) | 0.76 ± 0.03; (N=10; p=0.0010) | 0.72 ± 0.02; (N=11; p=0.0000) | 0.83 ± 0.01; (N=8; p=0.1944) | 0.78 ± 0.03; (N=10; p=0.0431) |
| **13 Hz Avg. NREM Spindle Duration (ms)** | 0.78 ± 0.02; (N=17) | 0.72 ± 0.02; (N=10; p=0.0067) | 0.72 ± 0.02; (N=11; p=0.0030) | 0.79 ± 0.02; (N=8; p=0.3575) | 0.75 ± 0.03; (N=10; p=0.2743) |
| **15 Hz Avg. NREM Spindle Duration (ms)** | 0.73 ± 0.02; (N=17) | 0.67 ± 0.02; (N=10; p=0.0041) | 0.68 ± 0.01; (N=11; p=0.0098) | 0.76 ± 0.02; (N=8; p=0.7603) | 0.72 ± 0.03; (N=10; p=0.4030) |
| **9 Hz Avg. NREM Spindle ISA$_s$** | 6818.38 ± 232.53; (N=16) | 6041.51 ± 241.32; (N=10; p=0.0031) | 6577.02 ± 273.24; (N=11; p=0.1612) | 6969.04 ± 151.96; (N=8; p=0.1668) | 6808.60 ± 411.37; (N=10; p=0.7215) |
| **11 Hz Avg. NREM Spindle ISA$_s$** | 8326.75 ± 367.33; (N=16) | 6990.02 ± 553.60; (N=10; p=0.0040) | 6678.89 ± 338.79; (N=11; p=0.0011) | 8659.29 ± 280.75; (N=8; p=0.2942) | 7755.10 ± 604.39; (N=10; p=0.1635) |
| **13 Hz Avg. NREM Spindle ISA$_s$** | 7991.64 ± 352.94; (N=17) | 6736.20 ± 463.87; (N=10; p=0.0083) | 7066.87 ± 432.18; (N=11; p=0.0167) | 8203.72 ± 408.56; (N=8; p=0.2399) | 7570.31 ± 581.36; (N=10; p=0.2895) |
| **15 Hz Avg. NREM Spindle ISA$_s$** | 7899.57 ± 358.50; (N=17) | 6396.22 ± 358.67; (N=10; p=0.0046) | 7138.03 ± 346.45; (N=11; p=0.0156) | 8083.87 ± 539.23; (N=8; p=0.3108) | 7459.35 ± 610.75; (N=10; p=0.2285) |

**Supplementary Table 4: Spindle duration and ISA$_s$ during NREM sleep across genotypes.** Mean ± SEM spindle duration are reported for each genotype. N represents number of animals analyzed for each genotype. p values are also reported for each genotype when compared to wildtype (*Cacna1i*$^{+/+}$). Homozygous *Cacna1i*$^{RH/RH}$ and *Cacna1i*$^{-/-}$ mice show significant reductions in spindle duration across all spindle frequencies. Colored cells indicate statistically significant differences when compared to the control group *Cacna1i*$^{+/+}$.
